# RhoGDIα regulates spermatogenesis through Rac1/cofilin/F-actin signaling

**DOI:** 10.1038/s42003-023-04579-7

**Published:** 2023-02-23

**Authors:** Haixia Zhu, Zongzhuang Wen, Aizhen Zhang, Dongyue Liu, Hongxiang Wang, Yin Cheng, Xing Yang, Yu Xiao, Jianyuan Li, Daqing Sun, Bin Wu, Jiangang Gao

**Affiliations:** 1 School of Life Science and Key Laboratory of the Ministry of Education for Experimental Teratology, Shandong University, Jinan, 250100 China; 2 Medical Science and Technology Innovation Center, Shandong First Medical University, Jinan, 250117 China; 3 Key Laboratory of Male Reproductive Health, National Health and Family Planning Commission, Beijing, 100081 China; 4 Department of Pediatric Surgery, Tianjin Medical University General Hospital, Tianjin, 300052 China; 5 Department of Reproductive Medicine, Central Hospital Affiliated to Shandong First Medical University, Jinan, 250013 China; 6 Cheeloo College of Medicine, Shandong University, Jinan, 250012 China

**Keywords:** Cell biology, Spermatogenesis

## Abstract

Spermatogenesis is an extremely complex process, and any obstruction can cause male infertility. *RhoGDIα* has been identified as a risk of male sterility. In this study, we generate *RhoGDIα* knockout mice, and find that the males have severely low fertility. The testes from *RhoGDIα*^*−/−*^ mice are smaller than that in WT mice. The numbers of spermatogonia and spermatocytes are decreased in *RhoGDIα*^*−/−*^ testis. Spermatogenesis is compromised, and spermatocyte meiosis is arrested at zygotene stage in *RhoGDIα*^*−/−*^ mice. Acrosome dysplasia is also observed in sperms of the mutant mice. At the molecular level, *RhoGDIα* deficiency activate the LIMK/cofilin signaling pathway, inhibiting F-actin depolymerization, impairing testis and inducing low fertility in mouse. In addition, the treatment of *RhoGDIα*^*−/−*^ mice with Rac1 inhibitor NSC23766 alleviate testis injury and improve sperm quality by inhibiting the LIMK/cofilin/F-actin pathway during spermatogenesis. Together, these findings reveal a previously unrecognized RhoGDIα/Rac1/F-actin-dependent mechanism involved in spermatogenesis and male fertility.

## Abstract (web summary)

RhoGDIα knockout mice show low male fertility and compromised spermatogenesis, with an underlying defect in F-actin cytoskeleton depolymerisation, which can be alleviated by inhibiting Rac1.

## Introduction

Spermatogenesis is a very complex process mainly including three biological processes: spermatogonia self-renewal and differentiation into spermatocytes, spermatocytes undergoing meiosis to produce haploid spermatids, and round sperm cells undergoing dramatic morphological transformation to form elongated spermatids^1^. Actin-related cytoskeletal changes affect many aspects of cellular activity, including morphogenesis, migration, phagocytosis, cytokinesis, and oocyte meiosis^2–4^. The regulation of the actin cytoskeleton plays a crucial role in spermatogenesis^5,6^. During mammalian spermatogenesis, three actin-rich structures, including ectoplasmic specialization (ES), manchette, and acroplaxome, play essential roles in sperm differentiation and morphological changes^7–9^. ES is of two types: basal ES and apical ES. The basal ES is an indispensable component of the blood-testis barrier^10^. The apical ES, which is present in the Sertoli cell-developing spermatid interface, is important for shaping the sperm head, sperm cell movement, and sperm release into the lumen of the seminiferous tubules^11^. Manchette consists of perinuclear ring, microtubules, and actin filaments^12,13^. F-actin- and microtubule-based transport provides some structural and functional support for nuclear shaping and flagellum formation^14,15^, and is vital in shaping the sperm head and sperm flagellum formation^16,17^. Acroplaxome comprising keratin 5 and F-actin anchors the developing acrosome to the elongating sperm nuclear envelope^13,18^ and cooperates with the manchette in the shaping of the spermatid head^19,20^.

Ras homolog (Rho)-specific guanine nucleotide dissociation inhibitors (RhoGDIs) are important regulators of the Rho family of small GTPases that control a wide range of cellular processes, including cell adhesion, migration, proliferation, and various diseases such as pancreatic, breast, bladder cancer and chronic nephrosis. RhoGDIs mainly binds to inactive Rho GTPases, prevents nucleotide exchange, and then acts as an inhibitory regulator of Rho GTPases. RhoGDIα is the most richly expressed and characteristic member of the family. The related research of RhoGDIα mainly focus on several Rho GTPases, such as the small GTPase Ras homolog gene family member A (RhoA), the small GTPase Ras-related C3 botulinum toxin substrate 1 (Rac1), Rac2, and cell division control protein 42 (Cdc42)^21^. RhoA activated Rho-associated protein kinase (ROCK), which can phosphorylate and activate LIM kinase (LIMK), while the activated LIMK phosphorylates and inhibits the activity of cofilin^22^. Rac1 binds to and activates p21-activated kinases (PAKs), which in turn phosphorylate and activate LIMK^23^. Further, LIMK regulates F-actin through cofolin^24^. Rac1 also plays a key role in regulating F-actin polymerization in cytoskeletal rearrangements^25^. Activated Rac1 can also bind to IRSp53, and IRSp53 binds to WAVE to form a trimolecular complex that stimulates the polymerization of new F-actin branches mediated by the Arp2/3 complex^26^. Cdc42 plays a role in regulating F-actin through its downstream Wiskott–Aldrich syndrome protein (WASP)^27^. WASP and N-WASP link Cdc42 to the Arp2/3 complex to promote F-actin polymerization^28^. Also, Cdc42 can bind to and activate PAKs^29^.

The dysregulation of Rho GTPases has been implicated in many human diseases^30^. However, the mechanism of Rho GTPases in regulating spermatogenesis remains to be elucidated. In this study, we successfully obtained *RhoGDIα*^*−/−*^ mice using CRISPR/Cas9 gene-editing technology and found that male *RhoGDIα*^*-−/−*^ mice were highly sub-fertile. Furthermore, we explored the molecular mechanisms, provided an evidence for the important role of Rac1 in male fertility. In summary, we suggested that RhoGDIα played an important role in maintaining testicular function and normal spermatogenesis through the Rac1/LIMK/cofilin signaling pathway, and providing a theoretical basis for developing drugs for male infertility.

## Results

### Deficiency of *RhoGDIα* caused male sub-fertility in mice

**Fig. 1:**
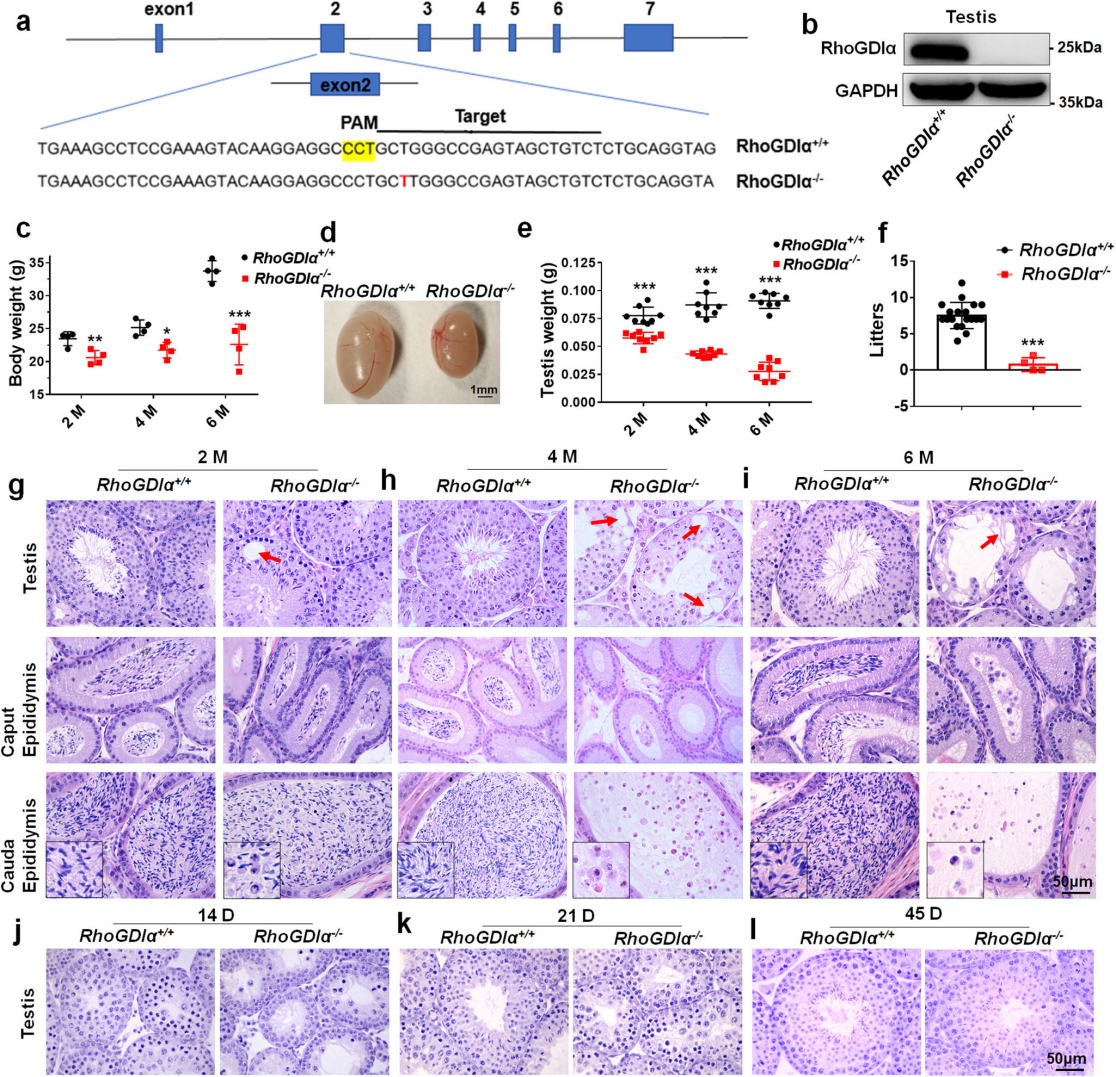
Testis showed progressive damage in *RhoGDIα*^−/−^ mice. **a** Schematic diagram of sgRNA targets for the RhoGDIα genome, and sgRNA target sites located in exon 2. **b** Western blot analysis was used to test the RhoGDIα protein, which was not expressed in *RhoGDIα*^*−/−*^ mice. **c** Body weight was checked in *RhoGDIα*^*+/+*^ and *RhoGDIα*^*−/−*^ male mice at 2 months (2 M), 4 months (4 M), and 6 months (6 M) of age. *n* = 4 biologically independent animals. **d** Photograph of *RhoGDIα*^*+/+*^ and *RhoGDIα*^*−/−*^ mouse testes. Scale bar = 1 mm. **e** Weight of mouse testes at 2 months, 4 months, and 6 months of age. *n* = 8 biologically independent animals. **f** Litters of mating WT female mice with *RhoGDIα*^*+/+*^ and *RhoGDIα*^*−/−*^ male mice for 3 months. *n* = 3 biologically independent male mice. **g**–**i** Histology of testis, caput, and cauda epididymis examined using H&E staining of mice at 2 months, 4 months, and 6 months of age. Red arrows indicate vacuolated seminiferous epithelium, while black boxed areas are higher-magnification images of cauda epididymis. **j**–**l** H&E staining result of *RhoGDIα*^*+/+*^ and *RhoGDIα*^*−/−*^ mouse testes at 14 days, 21 days, and 45 days of age, respectively. Scale bar = 50 μm. *P < 0.05, **P < 0.01, ***P < 0.001 *vs RhoGDIα*^*+/+*^. Data are presented as the mean ± standard error.

CRISPR/Cas9 gene-editing technology was used to delete *RhoGDIα* in mice. One single-guide RNA (sgRNA) was designed to target the specific sequence in exon 2 of the *RhoGDIα* gene (Fig. 1a). DNA sequencing results showed an insertion nearby the sgRNA target site (Supplementary Fig. 1a). The Western blot results showed that the RhoGDIα protein could no longer be detected in the *RhoGDIα*^*−/−*^ mice (Fig. 1b). We also examined the off-target effect of sgRNA. Four possible off-target genes were selected by sequence alignment: Cacna1d, Ripk4, Stat6, and Wdr60. DNA fragments nearby the four potential off-target sites were amplified and sequenced; no mutations were found in all the four potential off-target genes (Supplementary Fig. 1b-e). We tested other isoforms of RhoGDI, including *RhoGDIβ* and *RhoGDIγ*, and found that their transcript levels in the testis of *RhoGDIα*^*+/+*^ and *RhoGDIα*^*−/−*^ mice were similar (Supplementary Fig. 1f). These results indicated that *RhoGDIα*^*−/−*^ mice were successfully obtained by CRISPR/Cas9 gene-editing technology.

We found that self-bred offspring of heterozygous mutant mice conformed to Mendelian heredity law. The *RhoGDIα*^*−/−*^ male mice showed no abnormity in their daily behavior, but their body weight was smaller than that of the wild-type (WT) mice after 2, 4, and 6 months (Fig. 1c). Meanwhile, the size (Fig. 1d) and weight (Fig. 1e) of testes from adult *RhoGDIα*^*−/−*^ mice were significantly reduced as those of the WT mice. The reproductive ability of male *RhoGDIα*^*−/−*^ mice was significantly weakened. Only one *RhoGDIα*^*−/−*^ male mouse had offspring during the three months of co-caging with WT females, and the numbers of pups in two litters were 1 and 2 respectively (Fig. 1f). The reproductive ability of female *RhoGDIα*^*−/−*^ mice was also reduced. Only three litters (a total of five pups) were produced from two different *RhoGDIα*^*−/−*^ female mice (Supplementary Fig. 2a). Hematoxylin and eosin (H&E) staining results showed that the vacuolization appeared in the testes of 2-month-old *RhoGDIα*^*−/−*^ mice compared with *RhoGDIα*^*+/+*^ mice (Fig. 1g). The morphology of epithelial cells in the caput and cauda epididymis of *RhoGDIα*^*−/−*^ mice was normal, but many round sperms were observed in the cauda epididymis (Fig. 1g). In the 4-month-old *RhoGDIα*^*−/−*^ mice, severe vacuolization observed in seminiferous tubules, and the cells were lost severely. Only spermatocytes and round spermatids were observed, with no elongated spermatids in the testes (Fig. 1h). No normal sperms were found in the cauda epididymis, except round sperm cells and shed germ cells (Fig. 1h). At the age of 6 months, *RhoGDIα*^*−/−*^ mice had severe testicular degeneration (Fig. 1i). Most germ cells were lost, the epididymal epithelial cell morphology was still normal, and only round sperm cells and exfoliated germ cells were observed in the cauda epididymis. Meanwhile, the diameters of seminiferous tubules of *RhoGDIα*^*−/−*^ mice decreased compared with those of *RhoGDIα*^*+/+*^ mice (Supplementary Fig. 2b). Moreover, we explored whether *RhoGDIα* deficiency affected the development of testis. A large number of pachytene spermatocytes were observed in 14-day-old WT mice, but pachytene spermatocytes were observed widely until 21 days of age in *RhoGDIα*^*−/−*^ mice (Fig. 1j, k). At 45 days after birth, male mice are sexually mature. The sperms were closely arranged in the seminiferous tubule lumen in the WT mice, but few sperms were observed in the *RhoGDIα*^*−/−*^ mice testes (Fig. 1l). These results indicated that *RhoGDIα* knockout retarded testis development, resulted in progressive testes degeneration, and reduced fertility in male mice.

### Abnormal spermatogenesis occurred in *RhoGDIα*^*−/−*^ male mice

**Fig. 2:**
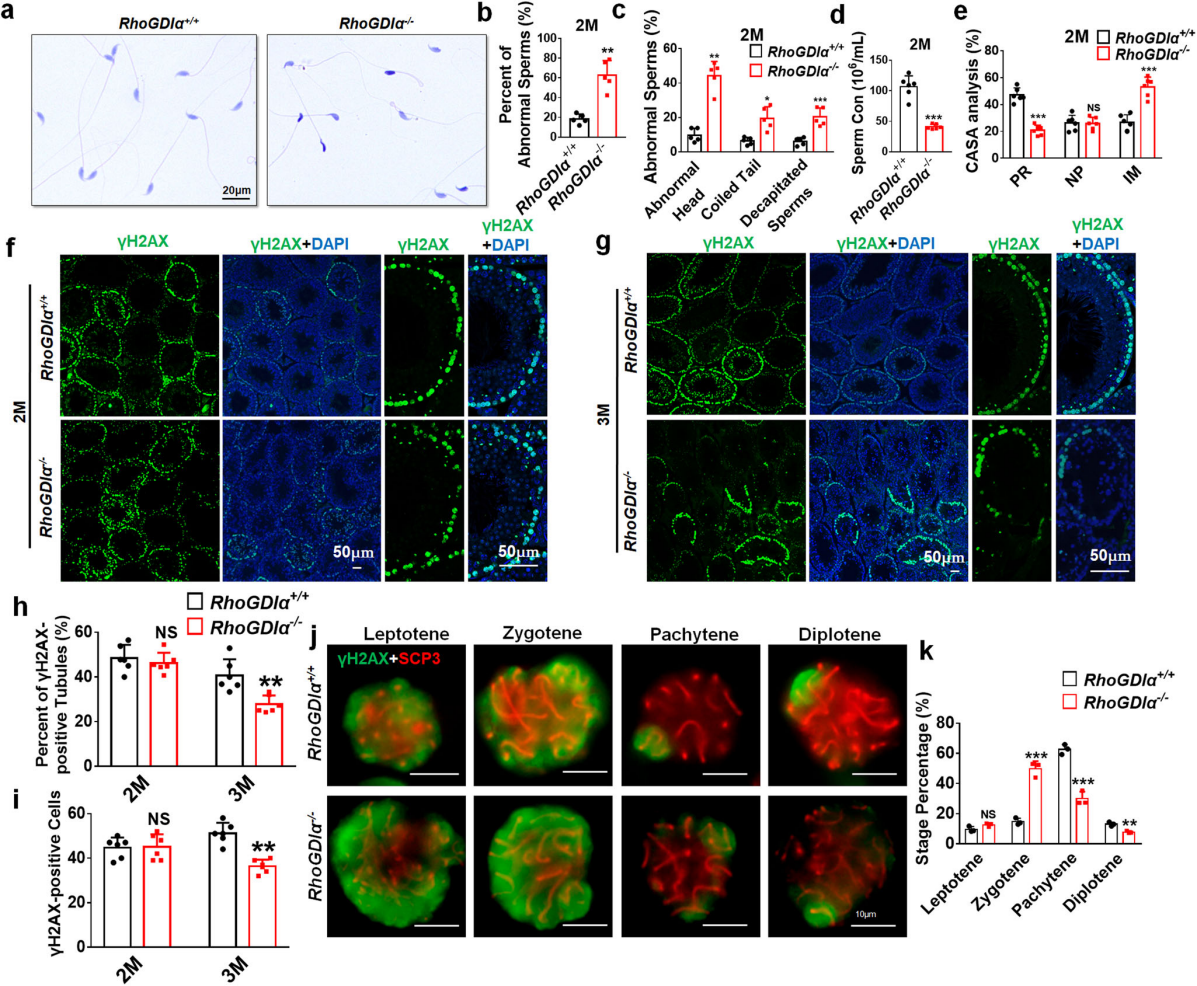
Evaluation of meiotic prophase and proliferation status of spermatocytes in *RhoGDIα*^*−/−*^ male mice. **a** Sperms from *RhoGDIα*^*+/+*^ and *RhoGDIα*^*−/−*^ mice at 2 months of age were stained with hematoxylin and eosin. Scale bar = 20 μm. **b** Percentage of total abnormal sperms and (**c**) sperms with different types of deformities from the caudal epididymis in *RhoGDIα*^*+/+*^ and *RhoGDIα*^*−/−*^ mice. **b**, **c** *n* = 5 biologically independent animals. **d** Sperm concentration in the caudal epididymis under the same conditions between *RhoGDIα*^*+/+*^ and *RhoGDIα*^*−/−*^ mice. **e** CASA analysis results of sperms in *RhoGDIα*^+/+^ and *RhoGDIα*^*−/−*^ mice. PR progressive motility, NP nonprogressive motility, IM immobilized sperms. **d**, **e** *n* = 6 biologically independent animals. **f**, **g** γH2AX-positive spermatocytes in the seminiferous epithelium from *RhoGDIα*^*+/+*^ and *RhoGDIα*^*−/−*^ mice at 2 months (**f**) and 3 months (**g**) of age. Scale bar = 50 μm. **h** Percentage of γH2AX-positive tubules and (**i**) γH2AX-positive cells in single tubules of *RhoGDIα*^*+/+*^ and *RhoGDIα*^−/−^ mice at 2 months and 3 months of age. *n* = 6 biologically independent animals. **j**, **k** SCP3 and γH2AX staining of spermatocytes from the testis of 3-month-old mice. *n* = 3 biologically independent animals. *P < 0.05, **P < 0.01, ***P < 0.001 *vs RhoGDIα*^+/+^. NS non-significant vs *RhoGDIα*^+/+^. Data are presented as the mean ± standard error.

The sperms from the cauda epididymis were subjected to H&E staining to observe the sperm morphology. At the age of 2 months, the number of abnormal sperms significantly increased in *RhoGDIα*^*−/−*^ mice compared with the WT mice (Fig. 2a, b), with abnormal head, coiled tail, and decapitated sperms, especially the proportion of sperms with abnormal head (Fig. 2c). The number of spermatozoa released from *RhoGDIα*^*−/−*^ cauda epididymis dramatically reduced (Fig. 2d). Moreover, as revealed by computer-assisted sperm analysis (CASA), the number of sperms with progressive motility decreased; meanwhile, and the number of immobilized sperms increased significantly in *RhoGDIα*^*−/−*^ mice (Fig. 2e). At the age of 3 months, only very few sperm have normal morphology in *RhoGDIα*^*−/−*^ mice (Supplementary Fig. 2c).

Proliferating cell nuclear antigen (PCNA) is a marker of DNA duplication, and it is used to examine the proliferation of spermatogonia and spermatocyte^31,32^. The number of PCNA-positive cells were found no obvious difference between 2-month-old *RhoGDIα*^*+/+*^ and *RhoGDIα*^*−/−*^ mice (Supplementary Fig. 2d), and decreased significantly in *RhoGDIα*^*−/−*^ mice after 3 months (Supplementary Fig. 2e). The differentiating spermatogonia were analyzed by c-KIT staining. Compared to the WT mice, the number of c-KIT-positive cells decreased in seminiferous tubules at the active stage of spermatogonia differentiation in the testis of 3-month-old *RhoGDIα*^*−/−*^ mice (Supplementary Fig. 2f, g). Although γH2AX is primarily a marker for DNA double-strand breaks, it can also be used to detect meiosis in the seminiferous tubules since it is widely expressed in the spermatocytes at the early stage of meiosis I^33^. Compared with *RhoGDIα*^*+/+*^ mice, the proportion of the seminiferous tubules that showed extensive staining of γH2AX did not change at 2-month *RhoGDIα*^*−/−*^ mice (Fig. 2f, h); and no difference was found in the number of γH2AX-positive cells in single tubules (Fig. 2f, i). However, in 3-month-old *RhoGDIα*^*−/−*^ mice, the tubules with large amounts of γH2AX decreased (Fig. 2g, h), and the number of γH2AX-positive cells in single tubules also significantly reduced (Fig. 2g, i). These results indicated that the deletion of *RhoGDIα* caused loss of spermatogonia and spermatocyte.

To determine the effects of *RhoGDIα* knockout on meiosis, we prepared chromosome spreads of spermatocytes isolated from the testis of 3-month-old mice, and performed immunostaining for SCP3 and γH2AX. (Fig. 2j). The percentage of pachytene spermatocytes was reduced from 62.64% in the *RhoGDIα*^*+/+*^ mice to 29.90% in the *RhoGDIα*^*−/−*^ mice, and the percentage of spermatocytes in the diplotene stage was also decreased. On the other hand, spermatocytes of *RhoGDIα*^*−/−*^ mice accumulated in the zygotene stage (*RhoGDIα*^*+/+*^ mice: 14.73%; *RhoGDIα*^*−/−*^ mice: 49.74%) (Fig. 2k). These results demonstrated that meiosis was arrested at the zygotene stage in the spermatocytes of *RhoGDIα*^*−/−*^ mice.

### Acrosome dysplasia after *RhoGDIα* deletion in male mice

**Fig. 3:**
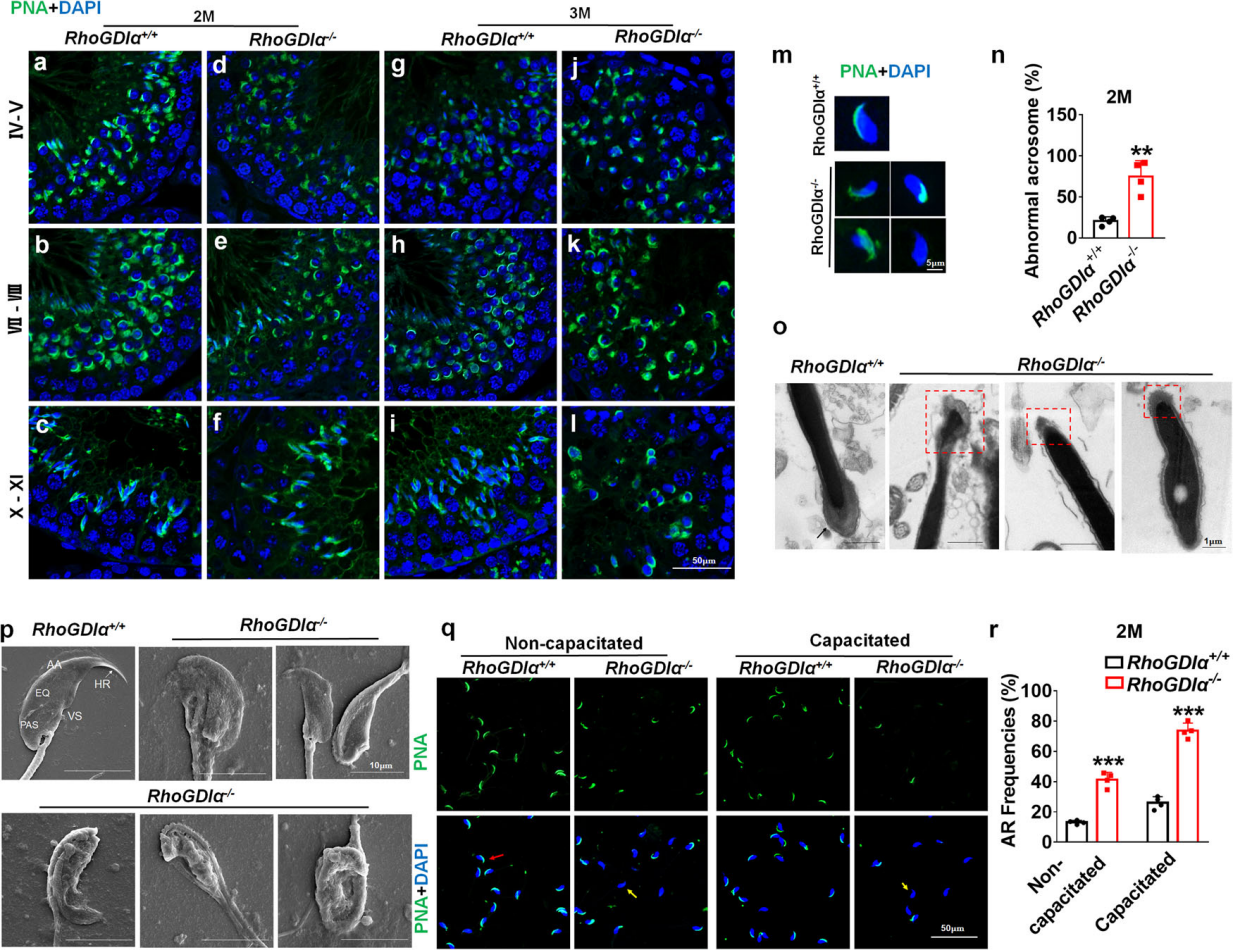
RhoGDIα is essential for acrosome morphology and function development. **a-l** FITC-labeled PNA was used to examine acrosomes at different developmental stages in *RhoGDIα*^+/+^ and *RhoGDIα*^*−/−*^ mice at 2 months and 3 months of age. Scale bar = 50 μm. **m** Acrosome morphology of sperms in the caudal epididymis from 2-month-old *RhoGDIα*^+/+^ and *RhoGDIα*^*−/−*^ mice. Scale bar = 5 μm. **n** Statistics of abnormal sperm proportion in mouse caudal epididymis. *n* = 4 biologically independent animals. **o** TEM images of the sperm acrosomes in 2-month-old *RhoGDIα*^+/+^ and *RhoGDIα*^*−/−*^. Scale bar = 1 μm. **p** SEM images of epididymal sperms from 2-months *RhoGDIα*^+/+^ and *RhoGDIα*^*−/−*^ mice. PAS Postacrosomal segment, EQ equatorial segment, AA anterior acrosome, HR hook rim, VS ventral spur. Scale bar = 10 μm. **q**, **r** Pictures and statistics of spontaneous AR frequencies in noncapacitated or capacitated sperms. Red arrow, normal acrosome; yellow arrow, spontaneous AR acrosome. Scale bar = 50 μm. *n* = 4 biologically independent animals. **P < 0.01, ***P < 0.001 *vs RhoGDIα*^*+/+*^. Data are presented as the mean ± standard error.

Sperm head malformation is closely related to acrosome development. We found the abnormal head sperms increased in *RhoGDIα*^*−/−*^ mice, then the acrosome development was assessed using FITC-labeled *Arachis hypogaea* (peanut) agglutinin (PNA) in the testis. In the seminiferous tubules of *RhoGDIα*^*+/+*^ mice, typical cap-shaped (stages IV-V) or crescent moon-shaped (stages VII–VIII) acrosomes were observed in round spermatozoa (Fig. 3a, b, g, h); the acrosomes were located at the top of the nuclei in the elongated spermatids (stages X–XI) (Fig. 3c, i). Besides, the sperms in the caudal epididymis showed that acrosomes were located at the top of sperm head and adhered to nuclei closely, showing a smooth crescent moon shape in *RhoGDIα*^*+/+*^ mice (Fig. 3m). In 2-month-old *RhoGDIα*^*−/−*^ mice, the spermatids with crescent-shaped acrosome were reduced (Fig. 3d–f), and the proportion of malformed acrosomes in the caudal epididymis increased, including fragmentation, diffusion, and missing acrosomes (Fig. 3m, n). In transmission electron microscopy (TEM) images, the acrosome of caudal epididymis sperms showed a full and regular shape in WT mice, as indicated by the black arrow (Fig. 3o). Sperms from *RhoGDIα*^*−/−*^ mice showed various defects in acrosomes, such as deformation and fragmentation as shown in red box (Fig. 3o). The scanning electron microscope (SEM) images showed that, the *RhoGDIα*-deficient sperms exhibited malformed head morphologies, deviating from the flat, crescent-shaped structures. Specific defects included the disorganized anterior acrosome and the noticeable absence of equatorial segment, post-acrosomal sheath, sharp hook rim, and ventral spur (Fig. 3p).

The sperms with acrosome dysplasia have a higher probability of spontaneous acrosome reaction (AR) before reaching the vicinity of the eggs, which prevents successful fertilization^34,35^. In *RhoGDIα*^*−/−*^ mice, the frequency of spontaneous AR increased significantly in the non-capacitated as well as capacitated sperm compared with those in the *RhoGDIα*^*+/+*^ mice (Fig. 3q, r). These results indicated that the morphology and function of acrosomes were abnormal in 2-month-old *RhoGDIα*^*−/−*^ mice.

The acrosomes with cap-shaped (stages IV-V) and crescent-shaped (stages VII–VIII) (Fig. 3j, k) could be observed in the testes of 3-month-old *RhoGDIα*^*−/−*^ mice, but the number of round spermatozoa severely reduced. When the seminiferous epithelium developed to stages X–XI, only a few spermatozoa were elongated (Fig. 3l), indicating that spermatogenesis was severely hindered at this stage.

### RhoGDIα regulated the LIMK/cofilin signaling pathway in testis and GC-1spg cells

**Fig. 4:**
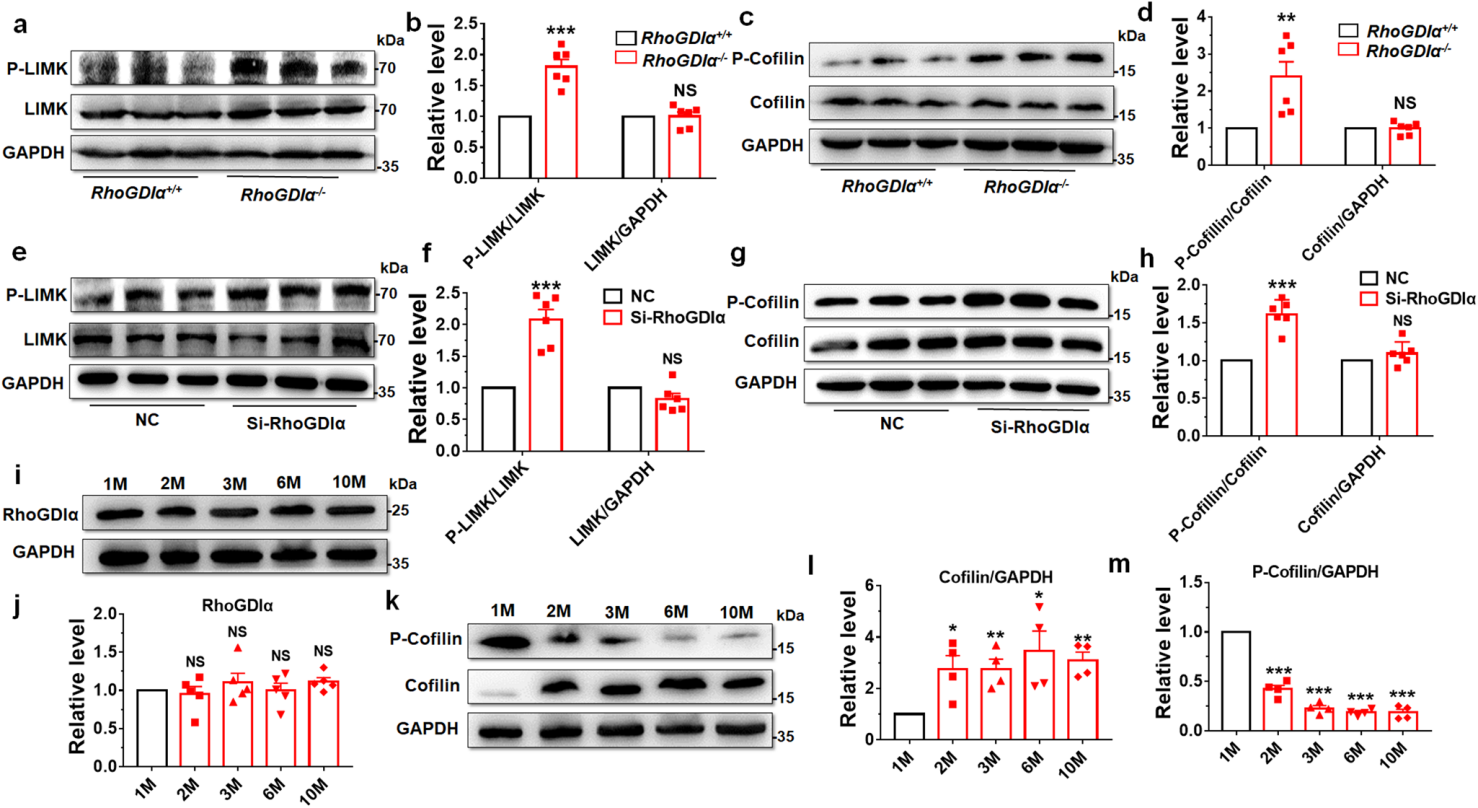
LIMK/cofilin signaling pathway was activated after knockout or knockdown of RhoGDIα. **a**–**d** The levels of P-LIMK, LIMK, P-cofilin, and cofilin were compared between *RhoGDIα*^*+/+*^ and *RhoGDIα*^−/−^ in mouse testes. *n* = 6 biologically independent animals. **e**, **f** GC-1spg cells were treated with Si- RhoGDIα for 48 h, and the protein was extracted to detect LIMK and its phosphorylation level. **g**, **h** Levels of P-cofilin and cofilin in GC-1spg cells were also tested after Si-RhoGDIα treatment. **f**, **h** *n* = 6 batches of cells in each group. **i**, **j** Protein level of RhoGDIα was detected by Western blot in WT mice at 1 month (1 M), 2 months (2 M), 3 months (3 M), 6 months (6 M), and 10 months (10 M) of age. *n* = 5 biologically independent animals. **k**–**m** Protein expression and phosphorylation of cofilin in WT mice of different ages. *n* = 4 biologically independent animals. *P < 0.05, **P < 0.01, ***P < 0.001 *vs RhoGDIα*^+/+^ or NC groups. NS non-significant vs *RhoGDIα*^+/+^ or NC groups. Data are presented as the mean ± standard error.

Rho family GTPase levels drastically reduced, but the levels of active Rho proteins (Rac1, Cdc42 and RhoA) increased after RhoGDIα depletion^36^. In this study, Rho GTPase levels also reduced after *RhoGDIα* deficiency in knockout mice or spermatogonia germ cell line (GC-1spg cells) (Supplementary Fig. 3a, b). And the active Rho proteins including Rac1 and RhoA were increased in *RhoGDIα*^*−/−*^ testis (Supplementary Fig. 3c, d). We observed the expression of LIMK and P-LIMK in the *RhoGDIα* knockout mouse testes. No difference in the total LIMK level was found, while P-LIMK expression was significantly upregulated in *RhoGDIα*^*−/−*^ testis (Fig. 4a, b). Phosphorylation-activated LIMK phosphorylated cofilin at Ser3, resulting in a significant increase in the phosphorylation level of cofilin in the testes of *RhoGDIα*^*−/−*^ mice, while the total cofilin level did not change (Fig. 4c, d). Moreover, immunohistochemical staining intuitively showed that P-cofilin expression increased in *RhoGDIα*^*−/−*^ mouse testes (Supplementary Fig. 4). Meanwhile, the pathway was also detected in GC-1spg cells. We designed three SiRNAs to knock down RhoGDIα. The RhoGDIα level was decreased remarkably at both mRNA and protein levels by Si-RhoGDIα-3 (Supplementary Fig. 5a, b). Therefore, Si-RhoGDIα-3 was used for follow-up experiments. The expression levels of LIMK and cofilin did not show any differences (Fig. 4e–h), but the phosphorylation levels of LIMK and cofilin significantly increased after Si-RhoGDIα transfection into GC-1spg cells compared with those in the negative control (NC) groups (Fig. 4e–h).

As the *RhoGDIα*^*−/−*^ mouse testis damaged progressively, the expression of RhoGDIα was observed in the WT mouse testes at different ages. Western blot results showed that RhoGDIα was expressed invariably in mice of different ages (Fig. 4i, j). Next, the cofilin and P-cofilin expression were tested. At the age of 1 month, the expression of cofilin was at a low level (Fig. 4k, l); it mainly existed in its inactive form P-cofilin (Fig. 4k, m). However, the expression of cofilin significantly increased and remained at a similar level at the age of 2–10 months (Fig. 4k, l), while the level of P-cofilin significantly decreased after 2 months (equivalent to about 16 years in humans^37^) and remained at a low level until the age of 10 months (Fig. 4k, m).

### *RhoGDIα* deficiency resulted in F-actin polymerization and apoptosis in testis and GC-1spg cells

**Fig. 5:**
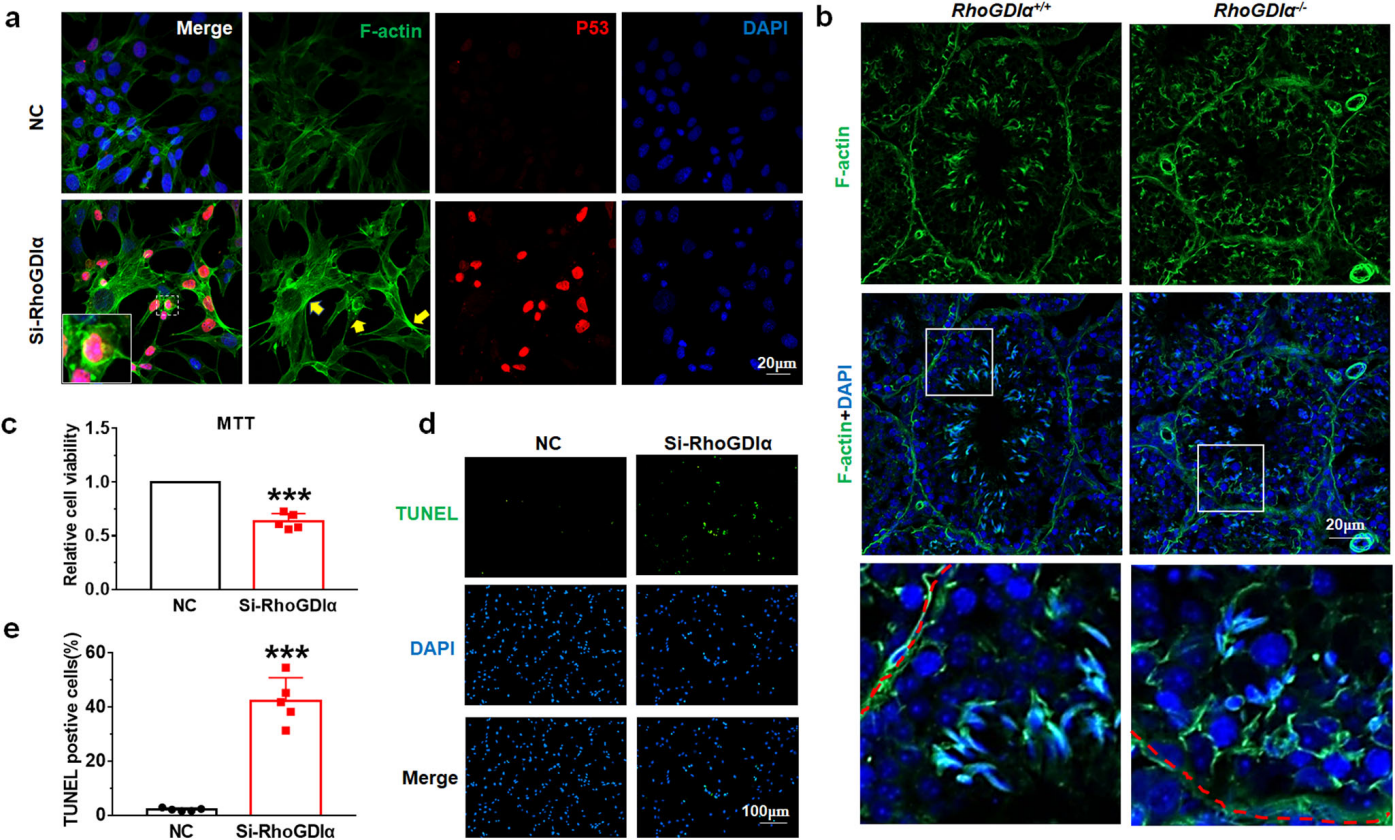
F-actin polymerization and apoptosis occurred after *RhoGDIα* deletion. **a** Staining of F-actin and P53 was achieved using the natural compounds phalloidin and secondary antibody conjugated with different fluorophores, respectively. The images in the white solid wire box are magnified images of the corresponding image. The yellow arrow points to the part where F-actin polymerization occurred abnormally. Scale bar = 20 μm. **b** F-actin expression in *RhoGDIα*^*+/+*^ and *RhoGDIα*^−/−^ mouse testes. The image on the last line is an enlarged view of the white box next to it. Scale bar = 20 μm. **c** MTT assay was used to test cell viability after transfection of Si-RhoGDIα into GC-1spg cells. **d**, **e** Apoptosis of GC-1spg cells after Si-RhoGDIα transfection was detected using TUNEL assay. **c**, **e** *n* = 5 batches of cells in each group. Scale bar = 100 μm. ***P < 0.001 *vs* NC group. Data are presented as the mean ± standard error.

Cofilin is required to control the F-actin depolymerization and assembly, which indicated that *RhoGDIα* deletion might adversely affect the depolymerization of F-actin. Phalloidin staining was used to observe F-actin to verify our hypothesis. In GC-1spg cells, F-actin exhibited abnormal aggregation in the Si-RhoGDIα groups compared with the NC group (Fig. 5a). Meanwhile, F-actin aggregated around germ cells abnormally in *RhoGDIα*^*−/−*^ mouse testes (Fig. 5b). We also isolated Sertoli cells to test F-actin levels, and detected aberrant expression of F-actin in the SOX9-positive Sertoli cells from *RhoGDIα*^*−/−*^ mice (as indicated by the red arrow) (Supplementary Fig. 6). These results indicating that the depolymerization of F-actin was inhibited after the deletion of *RhoGDIα*.

**Fig. 6:**
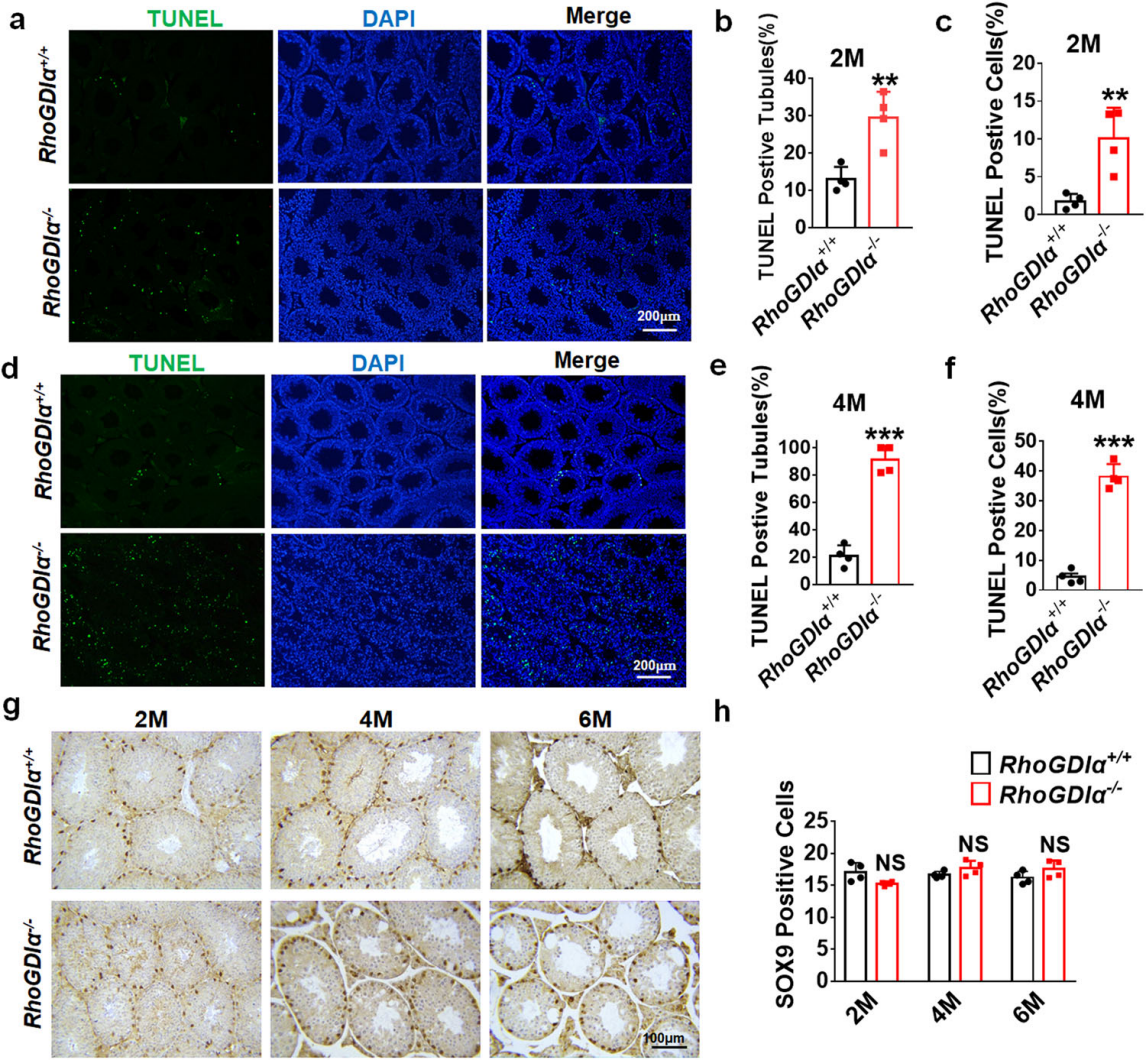
Apoptosis was detected in the testes of *RhoGDIα*^+/+^ and *RhoGDIα*^*−/−*^ mice. **a** Testicular apoptosis was detected by TUNEL assay at the age of 2 months. Scale bar = 20 μm. **b**, **c** Proportion of TUNEL-positive tubules and positive cells in 2-month-old mouse testes. *n* = 4 biologically independent animals. **d** Representative diagram of testicular apoptosis in mice at 4 months of age. Scale bar = 20 μm. **e**, **f** Statistical results of the TUNEL experiment in Fig. 4d. *n* = 4 biologically independent animals. **g** Representative images of the SOX9 immunohistochemistry of *RhoGDIα*^*+/+*^ and *RhoGDIα*^*−/−*^ mice at 2 months (2 M), 4 months (4 M), and 6 months (6 M) of age. **h** Number of SOX9-positive cells in mouse testes. *n* = 4 testes from 4 biologically independent animals, more than 20 tubules were counted in each testis. **P < 0.01, ***P < 0.001 *vs RhoGDIα*^*+/+*^, NS non-significant *vs RhoGDIα*^*+/+*^. Data are presented as the mean ± standard error.

Beside this, the 3‐(4, 5‐dimethylthiazol‐2‐yl)‐2, 5‐diphenyltetrazolium bromide (MTT) experiment showed that the GC-1spg cell viability decreased after RhoGDIα was knocked down (Fig. 5c). P53 expression was tested to examine whether cell apoptosis occurred in GC-1spg cells. Immunofluorescence detection showed that Si-RhoGDIα increased p53 expression and promoted its nuclear accumulation (Fig. 5a). Terminal deoxynucleotidyl transferase‐mediated dUTP‐biotin nick-end labeling (TUNEL) assay was performed to observe the apoptosis. The proportion of TUNEL-positive GC-1spg cells increased after Si-RhoGDIα treatment (Fig. 5d, e). In testis, the results showed that both the proportion of TUNEL-positive seminiferous tubules (Fig. 6a, b) and the number of apoptotic cells in these apoptosis tubules (Fig. 6a, c) increased in 2-month-old *RhoGDIα*^*−/−*^ mice. In 4 months, apoptosis was more severe in *RhoGDIα*^*−/−*^ mice (Fig. 6d). The percentage of apoptotic seminiferous tubules increased to 91% compared with 29% at 2 months (Fig. 6b, e), and the percentage of apoptotic cells in each seminiferous tubule increased to 38% (Fig. 6f). SOX9-marked Sertoli cells were counted, and the result showed that no difference was found in the Sertoli cell count of *RhoGDIα*^*−/−*^ mice after 2 months, 4 months, and 6 months compared with *RhoGDIα*^*+/+*^ mice (Fig. 6g, h). This result indicated that *RhoGDIα* deletion did not cause the loss of Sertoli cells.

### Rac1 or ROCK inhibitor alleviated GC-1spg cell injury induced by Si-RhoGDIα

**Fig. 7:**
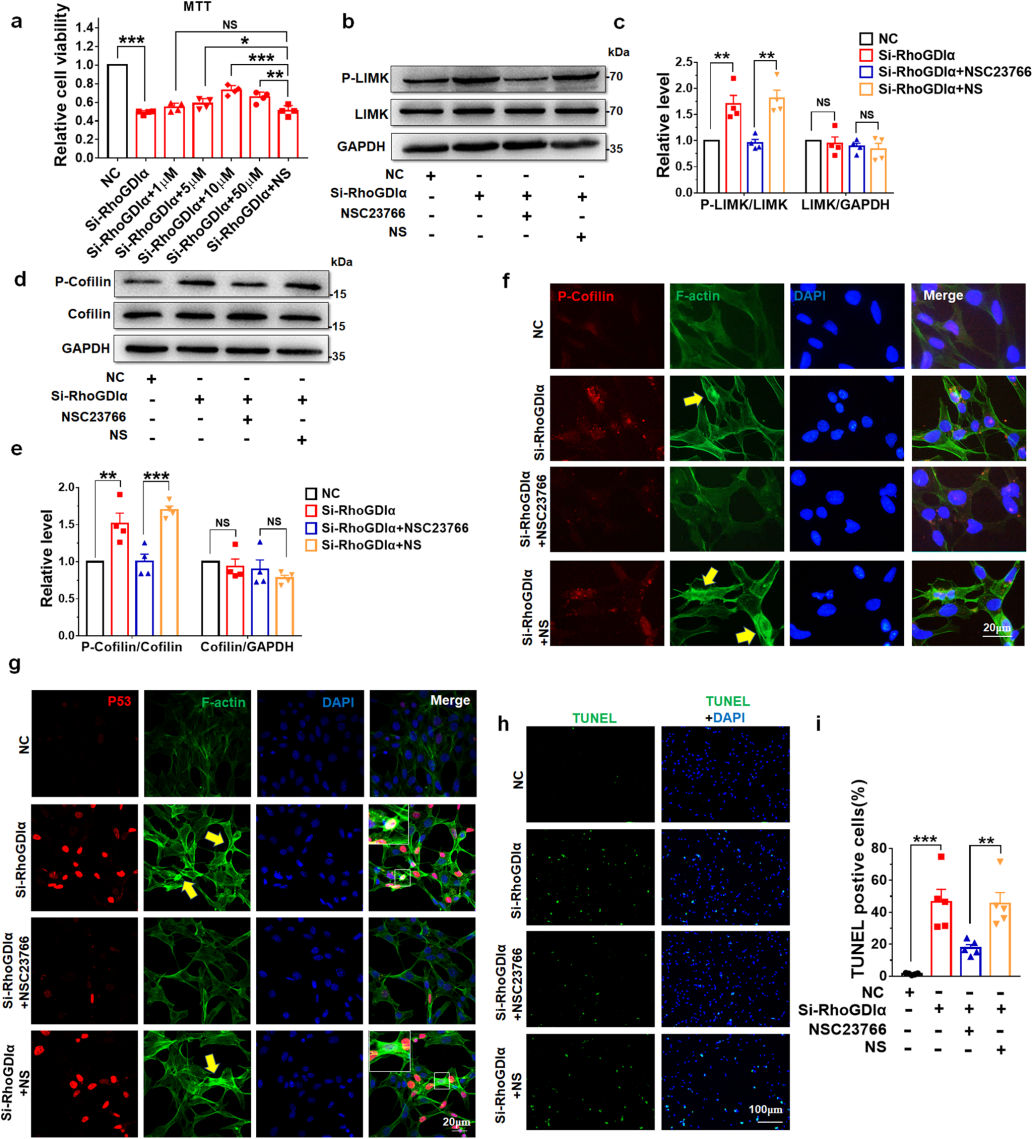
NSC23766 mitigated the effects of RhoGDIα knockdown on GC1-spg cells. **a** Optimum concentration of NSC23766 to improve cell viability was evaluated by MTT assay. 1 μM, 5 μM, 10 μM, and 50 μM represent the concentration of NSC23766, and NS represents normal saline. **b**, **c** GC-1spg cells were treated with 10 μM NSC23766 for 48 h after transfection with Si-RhoGDIα, and then P-LIMK and LIMK levels were tested. **d**, **e** Cofilin and its phosphorylation level were tested after NSC23766 treatment. **a**, **c,** and **e** *n* = 4 batches of cells in each group. **f** Immunofluorescence results of P-cofilin and F-actin co-staining in GC-1spg cells. Scale bar = 20 μm. **g** Immunofluorescence staining of P53 and F-actin in GC1-spg cell transfection with Si-RhoGDIα and then treatment with NSC23766. Scale bar = 20 μm. The images in the white solid wire box are magnified images of the corresponding white-dotted box. The yellow arrow indicates abnormal aggregation of F-actin. **h**, **i** TUNEL assay was used to test GC1-spg cell apoptosis. Scale bar = 100 μm. *n* = 5 batches of cells in each group. *P < 0.05, **P < 0.01, ***P < 0.001, NS non-significant. Data are presented as the mean ± standard error.

NSC23766 (a unique Rac-specific inhibitor) was used to inhibit the activity of Rac1 in GC-1spg cells so as to suggest that RhoGDIα regulated the polymerization and depolymerization of F-actin via the Rac1/LIMK/cofilin signaling pathway. NSC23766 could effectively improve cell viability, and the 10 µM NSC23766 dose showed better effects, which was selected for further experiments (Fig. 7a). The data of this study showed that NSC23766 repressed the elevated levels of the P-LIMK and P-cofilin induced by Si-RhoGDIα (Fig. 7b–e) and reduced the abnormal aggregation of F-actin after Si-RhoGDIα treatment (Fig. 7f, g). After RhoGDIα was knocked down, the aggregation of F-actin was most obvious in cells with high expression of P-cofilin (Fig. 7f). This further suggested that the abnormal accumulation of F-actin was mostly influenced by increased P-cofilin under RhoGDIα knockdown. Moreover, NSC23766 alleviated the nuclear translocation of P53 under Si-RhoGDIα, as shown by P53 immunofluorescence results, the number and brightness of P53 positive nuclei decreased (Fig. 7g), and the number of TUNEL-positive cells reduced markedly by the inhibition of Rac1 after RhoGDIα knockdown (Fig. 7h, i). These results indicated that NSC23766 alleviated GC-1spg cell injury caused by RhoGDIα deficiency though LIMK/cofilin pathway.

Besides Rac1, the Rho family GTPase RhoA and its downstream effector ROCK also controlled many different cellular processes^38,39^. In GC-1spg cells, Y-27632 (ROCK inhibitor) improved cell viability after RhoGDIα knockdown (Supplementary Fig. 7a). The phosphorylation levels of LIMK and cofilin were also reduced by Y-27632 after the transfection of Si-RhoGDIα (Supplementary Fig. 7b, c). Besides, the inhibitor of ROCK repressed the extensive F-actin polymerization (Supplementary Fig. 7d) and cell apoptosis (Supplementary Fig. 7e, f) induced by the knockdown of RhoGDIα.

### Rac1 inhibitor improved sperm quality in *RhoGDIα*^*−/−*^ mice

**Fig. 8:**
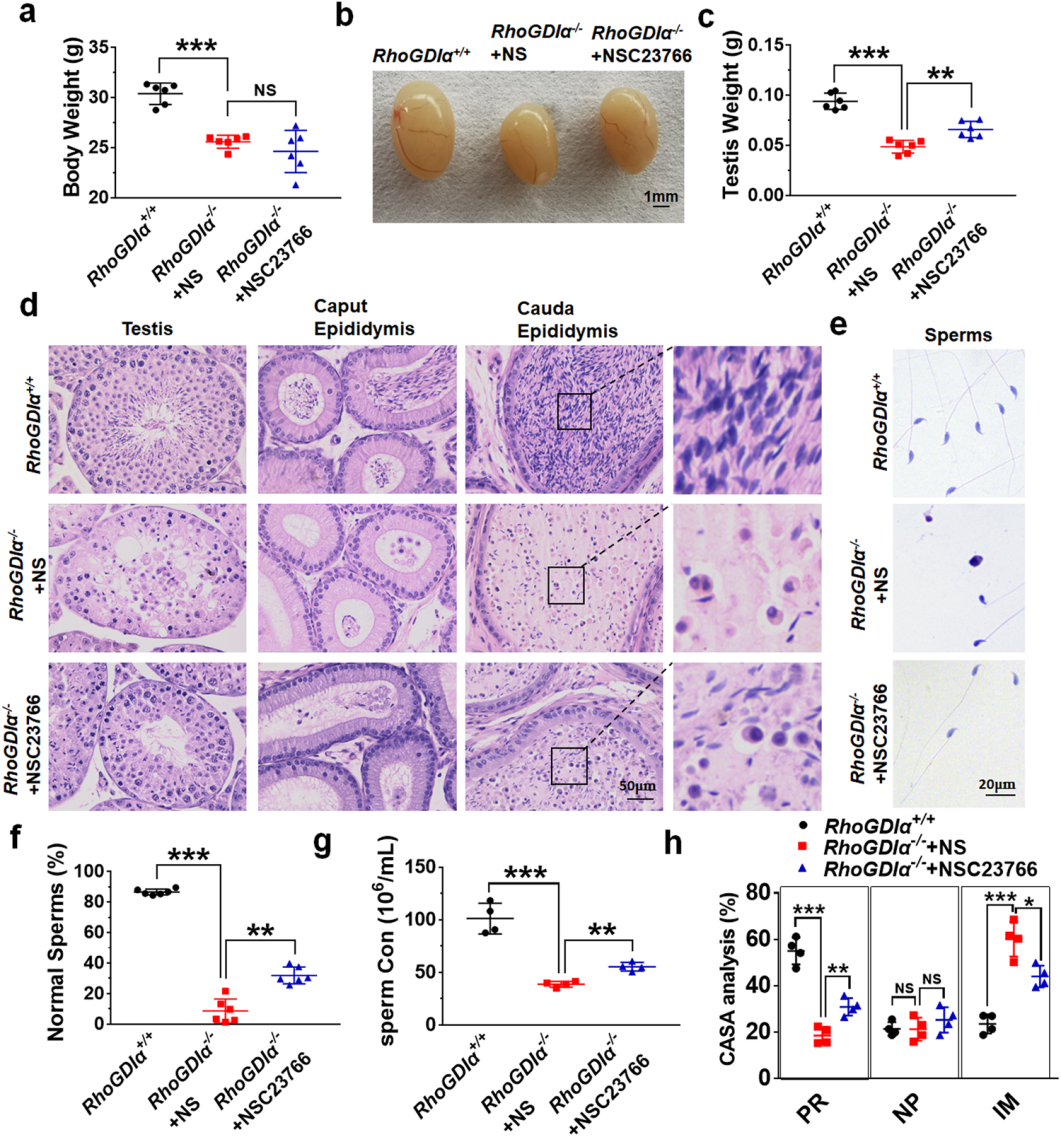
NSC23766 improved the sperm quality of *RhoGDIα*^*−/−*^ mice. **a** Body weight of *RhoGDIα*^*+/+*^ mice, *RhoGDIα*^*−/−*^ mice, and *RhoGDIα*^*−/−*^ mice with NSC23766. **b**, **c** Picture and weight of mouse testes. Scale bar = 1 mm. **a**, **c** *n* = 6 biologically independent animals. **d** Histological morphology of testis in mice after NSC23766 administration. The image on the right is the larger version in the black box of cauda epididymis. Scale bar = 50 μm. **e** H&E staining of sperms from the caudal epididymis of *RhoGDIα*^*+/+*^ mice, *RhoGDIα*^*−/−*^ mice, and *RhoGDIα*^*−/−*^ mice with NSC23766. Scale bar = 20 μm. **f** Proportion of morphologic normal sperms was calculated based on H&E staining. *n* = 6 biologically independent animals. **g** Sperm concentration from the caudal epididymis under the same treatment conditions. **h** CASA analysis was used to measure sperm motility after NSC23766 treatment in *RhoGDIα*^*−/−*^ mice. PR progressive motility, NP nonprogressive motility, IM immobilized sperm. **g**, **h** *n* = 4 biologically independent animals. *P < 0.05, **P < 0.01, ***P < 0.001, NS non-significant. Data are presented as the mean ± standard error.

Then whether NSC23766 could improve the sperm quality and degeneration of spermatogenic epithelium in *RhoGDIα*^*−/−*^ mice was examined. NSC23766 or normal saline (NS) was injected into a micro-sustained-release pump, and the pump was administered to *RhoGDIα*^*−/−*^ mice at the age of 8 weeks. After 4 weeks of medication, the body weight of *RhoGDIα*^*−/−*^ mice with the NSC23766 pump did not increase compared with that of *RhoGDIα*^*−/−*^ mice treated with NS (Fig. 8a). However, NSC23766 ameliorated the reduction in testis volume and weight caused by *RhoGDIα* knockout (Fig. 8b, c). The administration of NSC23766 reduced the histological damage in testis, concomitantly with improving sperm morphology and concentration in the cauda epididymis (Fig. 8d). Sperms released from the caudal epididymis were collected and stained with H&E to reveal their morphology clearly (Fig. 8e). Statistics showed that the proportion of normal morphology sperms was increased after NSC23766 treatment (Fig. 8f). The reduced sperm concentration in *RhoGDIα*^*−/−*^ mice was substantially ameliorated by NSC23766 treatment (Fig. 8g). CASA analysis also showed that progressive motility of sperms improved, and the number of immobilized sperms decreased by NSC23766 in *RhoGDIα*^*−/−*^ mice compared with *RhoGDIα*^*−/−*^ mice after treatment with NS (Fig. 8h). These results indicated that NSC23766 improved sperm quality in *RhoGDIα*^*−/−*^ mice.

### NSC23766 reduced testicular injury by inhibiting LIMK/cofilin pathway in *RhoGDIα*^*−/−*^ mice

**Fig. 9:**
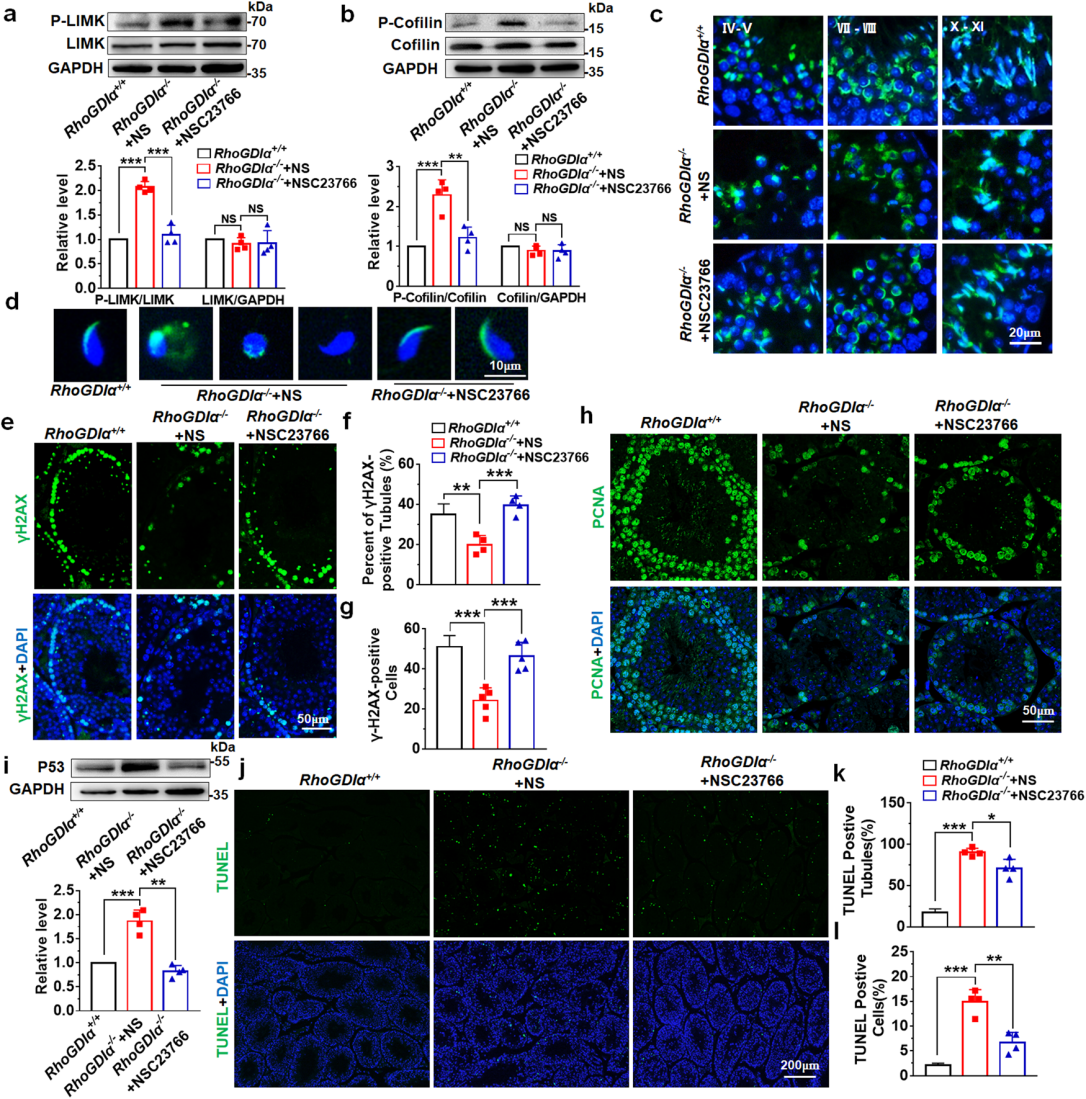
NSC23766 ameliorated testis injury in *RhoGDIα*^***−/−***^ mice. **a** After NSC23766 treatment of 8-week-old *RhoGDIα*^*−/−*^ mice for 4 weeks, LIMK and its phosphorylation levels were detected by Western blot analysis. **b** Levels of P-cofilin and cofilin of testis were also tested in *RhoGDIα*^*+/+*^ mice, *RhoGDIα*^*−/−*^+NS mice, and *RhoGDIα*^*−/−*^+NSC23766 mice. **a**, **b** *n* = 4 biologically independent animals. **c** Acrosomes were tested in the *RhoGDIα*^*−/−*^ mouse testes after administration of NSC23766. Scale bar = 20 μm. **d** FITC-labeled PNA was used to display the acrosomal morphology of sperms. Scale bar = 10 μm. **e**–**g** γH2AX was used to examine the effect of NSC23766 on spermatocyte meiosis in *RhoGDIα*^*−/−*^ mouse testes. Scale bar = 50 μm. **f** *n* = 4 biologically independent animals. **g** *n* = 5 biologically independent animals. **h** Role of Rac1 inhibitor NSC23766 on the proliferation of testicular germ cells in *RhoGDIα*^*−/−*^ mice was tested by immunofluorescence of PCNA. Scale bar = 50 μm. **i** Expression of P53 protein was detected in testes using GAPDH as a loading control. *n* = 4 biologically independent animals. **j**–**l** TUNEL assay was used to test the apoptosis level of testis in *RhoGDIα*^+/+^ mice, *RhoGDIα*^*−/−*^ +NS mice, and *RhoGDIα*^*−/−*^ +NSC23766 mice. Scale bar = 200 μm. *n* = 4 biologically independent animals. *P < 0.05, **P < 0.01, ***P < 0.001, NS non-significant. Data are presented as the mean ± standard error.

This study investigated whether NSC23766 functioned through the LIMK/cofilin signaling to improve sperm quality in *RhoGDIα*^*−/−*^ mice. Western blot results showed that the phosphorylation level of LIMK increased after *RhoGDIα* deletion, which was inhibited by NSC23766 administration (Fig. 9a). Subsequently, the phosphorylation of cofilin was inhibited by NSC23766 in *RhoGDIα*^*−/−*^ testes (Fig. 9b). In the testis, the numbers of both round spermatozoa and elongating spermatids with normal acrosomes were increased under NSC23766 treatment (Fig. 9c). The sperms in the caudal epididymis also exhibited acrosomes with normal shape after NSC23766 treatment in *RhoGDIα*^*−/−*^ mice (Fig. 9d). Besides, NSC23766 increased the numbers of both the γH2AX-positive seminiferous tubules (Fig. 9f and Supplementary Fig. 8) and γH2AX-positive cells of single tubules (Fig. 9e, g) in *RhoGDIα*^*−/−*^ male mice. The immunofluorescence results of PCNA showed that the proliferating germ cells significantly increased after NSC23766 treatment in *RhoGDIα*^*−/−*^ mice (Fig. 9h). Also, NSC23766 protected testicular cells from apoptosis injury, which was evident by the decreased expression level of P53 protein (Fig. 9i) and reduced number of TUNEL-positive cells (Fig. 9j–l) in *RhoGDIα*^*−/−*^+NSC23766 groups. In a word, the aforementioned results showed that the Rac1 inhibitor alleviated the testis injury in *RhoGDIα*^*−/−*^ mice.

## Discussion

RhoGDIα was identified as a down-regulated regulator of Rho family GTPase. It is involved in various biological processes, although its role in spermatogenesis is not completely clear. To this end, we generated *RhoGDIα*^*−/−*^ mice, which are highly sub-fertile. We explored the mechanisms underlying male infertility in these mice, and identified molecular targets that may be utilized in alleviating testis injury.

The RhoGDI family of proteins control Rho GTPases by inhibiting the dissociation of GDP (inactive form), promoting the interaction of the active Rho GTPases with their effector proteins, targeting the Rho GTPases to the subcellular compartment, and preventing hydrolysis of the bound GTP^40^. In RhoGDI family, RhoGDIα is widely expressed in many tissues^41,42^, and plays an important role in cell division, morphology and migration. It primarily exerts its effects through Rho GTPases-mediated depolymerization and polymerization of F-actin^43^. The optimal level of RhoGDIα is necessary for maintaining normal tissue morphogenesis and development. For instance, overexpression of *RhoGDIα* in a transgenic mouse model inhibited cardiomyocyte proliferation, and disrupted cardiac morphogenesis^44^. On the other hand, *RhoGDIα*^*−/−*^ mice have been shown to develop progressive renal disease and die due to kidney failure within a year^45^.

In our study, the *RhoGDIα*^*−/−*^ mice weighed less and had smaller kidney and testis compared to their wild-type counterparts, which is consistent with previous reports^45,46^. These results indicated that RhoGDIα plays an essential role in organ growth and development. We focused on the function of RhoGDIα in spermatogenesis. We found that the numbers of spermatogonia and spermatocytes were significantly decreased, and the development of seminiferous tubule was delayed in the *RhoGDIα*^*−/−*^ mice. In addition, the testicular defects in these became more severe with aging, which can be attributed to the age-dependent depletion of spermatogonia. Abnormal spermatogenesis and acrosome dysplasia were also observed in the testis of *RhoGDIα*^*−/−*^ mice. The molecular mechanisms were further explored. A previous study showed that, in *RhoGDIα*^*−/−*^ lung, RhoA activity is increased while that of Rac1 is unaffected^47^. Furthermore, there are reports that, in the renal, *RhoGDIα* deficiency increased the level of active Rac1 but not that of RhoA^46,48,49^. Thus, the regulatory effects of *RhoGDIα* are highly complex and involve multiple signaling pathways, as well as tissue-specific molecular mechanisms. We found that the activity of Rac1 and RhoA were increased in the testis of *RhoGDIα*^*−/−*^ mice, and the LIMK/cofilin pathway was also activated.

The increased level of P-cofilin disrupted the depolymerization of F-actin in the testis of *RhoGDIα*^*−/−*^ mice. The dynamic regulation of F-actin polymerization and disassembly controls cell division and morphology^50^. F-actin disorders are associated with a reduced number of germ cells, meiosis arrest at the zygotene stage, and acrosome dysplasia. In addition, the F-actin in Sertoli cells also plays an important role in spermatogenesis and male infertility^51^. We detected aberrant F-actin depolymerization in the Sertoli cells isolated from *RhoGDIα*^*−/−*^ mice, which was indicative of a dysfunctional status. Furthermore, we also observed some round spermatozoa shedding in the cauda epididymis of the *RhoGDIα*^*−/−*^ mice, which may be related to the dysfunction of Sertoli cells^51,52^. Therefore, *RhoGDIα* deletion phenotypes may be caused by both the defects in germ cells as well as F-actin disorders in Sertoli cells. However, the specific function of *RhoGDIα* in germ cells and Sertoli cells remains unclear. To clarify this point, we will construct Floxed mice and crossbreed them with AMH-Cre (Sertoli cell-specific expression) or Stra8-Cre (spermatogonia-specific expression) mice, in order to explore the specific defects in Sertoli cells and germ cells caused by *RhoGDIα* deletion.

We used GC-1spg cells to identify potential molecular targets for reversing the testicular damage caused by *RhoGDIα* depletion. Several studies have used this cellular model to elucidate the function of spermatogonia^53–55^. We tested the effects of Rac1 inhibitor (NSC23766) and Rock1 inhibitor (Y-27632) in GC-1spg cells transfected with Si-RhoGDIα. Based on the stable effects of NSC23766 on GC-1spg cells, we treated 8-week-old *RhoGDIα*^*−/−*^ male mice with different concentrations (2.5, 5, 8 and 10 mg/kg/d) of the drug. We found that the concentrations of 5–8 mg/kg/d alleviated testis injury and improved sperm quality in *RhoGDIα*^*−/−*^ mice. Although a recent report showed that GC-1spg cells are more likely to be the derivatives of Leydig cells (10.1101/2021.01.07.425754), this does not affect our conclusions since they are mainly derived from testis and sperm. Furthermore, the GC-1spg cells merely reinforce the results of in vivo experiments, which suggests that regulating Rho GTPases activity may be a potential therapy for the male sterility.

The male *RhoGDIα*^*−/−*^ mice showed impaired testis and massive proteinuria, and died due to renal failure within a year^45^. This is consistent with the report that mutations in the human homolog of *RhoGDIα* (*ARHGDIA*) can cause nephrotic syndrome^49^. It is unclear that whether *RhoGDIα* mutations manifest as abnormal reproductive phenotypes, and it is worth testing reproductive function in mice harboring these mutations. *ARHGDIA* mutations result in the failure to regulate the migration of human podocytes, and are related to the activation of Rac1 and the regulation of actin^49,56,57^. This is in line with our findings in the testis of *RhoGDIα*^*−/−*^ mice. It will be worth analyzing whether men with fertility issues harbor mutations in *ARHGDIA*.

In summary, this is the first study reporting the function of *RhoGDIα* in spermatogenesis and fertility. We found that genetic ablation of *RhoGDIα* activated the LIMK/cofilin signaling pathway, inhibited F-actin depolymerization and induced germ cell apoptosis. The loss of germ cells in the testis of *RhoGDIα*^*−/−*^ mice led to extensive vacuolation. During sperm head shaping, the abnormal F-actin dynamic also caused acrosome dysplasia, which resulted in malformed sperm head and acrosome. Furthermore, a Rac1 inhibitor alleviated the testicular defects in *RhoGDIα*^*−/−*^ mice through the Rac1/cofilin/F-actin pathway, indicating a possible molecular target for treating infertility in men.

## Methods

### Ethical statement

The health C57BL/6 mice (≤6 months old) were kept under standard conditions for animals (Temperature, 21 ± 1 °C; Humidity, 50–60%) and a light/dark cycle of 12/12 h. All animal experiments are conducted in accordance with the approval of the Ethics Committee of Shandong University (Jinan, China), and animal management is strictly in accordance with the Animal Ethics Standards of Shandong University.

### Animals

*RhoGDIα*^*−/−*^ mice were generated using the CRISPR/Cas9 technology. The sgRNA target sequence was as follows: 5′- AGACAGCTACTCGGCCCAGC −3′. The *hCas9* mRNA and sgRNAs were produced by in vitro transcription using an mMESSAGE mMACHINE T7 kit (Am1345; AmbionInc, Austin, TX, USA). WT C57BL/6 superovulated females were mated with WT males to obtain zygotes for *Cas9* mRNA (50 ng/ml) and sgRNA (25 ng/ml) injection. In the newborn founders, the genomic DNA surrounding the sgRNA target sites was amplified by polymerase chain reaction (PCR) (Forward primers: 5′- GCTGTCACAGTCCTGCTCAT -3′; and Reverse primers, 5′- GCACTTCCCCTCTTCTCCAC -3′), and the PCR products were sequenced to identify the mutations. NSC23766 (Aladdin, China) was subcutaneously infused via an osmotic minipump (Alza, USA) into *RhoGDIα*^*−/−*^ male mice at 8 weeks of age for 4 weeks. Our study revealed that NSC23766 at a dose of 5–8 mg kg^−1^ d^−1^ ameliorated testicular injury.

### Fertility test

For assessing the reproductive ability, 2-month-old *RhoGDIα*^*+/+*^ or *RhoGDIα*^*−/−*^ male mice were caged with 2-month-old WT female mice in a ratio of 1:2 and allowed to mate naturally for 3 months. Three male mice were tested. And WT or *RhoGDIα*^*−/−*^ female mice were caged with WT male mice at a ratio of 2:1 for 3 months to test the reproductive ability of *RhoGDIα*^*−/−*^ female mice. Six female mice were used in each group. The number of pups per litter was observed and recorded. Three mating cages were set for each genotype.

### Computer-assisted sperm analysis

The sperm from the caudal epididymis was analyzed. The cauda epididymis of adult mice was removed and washed with phosphate-buffered saline (PBS), and then placed in an M2 medium (Sigma Aldrich, USA). The incisions were made into the caudal epididymis and placed at 37 °C under 5% CO_2_ for 30 min to make sperm swim out from the caudal epididymis incision. The tissue fragments were removed and supplemented with M2 medium to 500 μL. Then, 10 μL of the fluid was placed on the counting slide to assess sperm motility using a computer-assisted sperm analysis system (Tsinghua Tongfang Co., LTD., China). More than 200 sperms were counted for each test. Three counting slides were tested and averaged in each mouse.

### Histological analysis

The testis and epididymis of *RhoGDIα*^*+/+*^ or *RhoGDIα*^*−/−*^ mice were fixed in Bouin’s solution overnight at 4 °C. After a series of gradient dehydration and embedding in paraffin, the tissues were cut into sections of 4 μm thickness. According to standard procedures, the tissue sections were stained with H&E. Finally, the stained sections were transparent in xylene and sealed with neutral gum. The histological structure was observed under an optical microscope (Nikon YS100, Japan).

### Immunofluorescence staining

The testes were fixed in 4% paraformaldehyde (PFA) overnight at 4 °C. They were next dehydrated and embedded in paraffin or Tissue-Tek Optimal Cutting Temperature (OCT) Compound (SAKURA, USA). The paraffin sections were dewaxed and rehydrated, and the frozen sections were washed with PBS. Subsequently, antigen repair was performed with 0.01% sodium citrate buffer (pH 6.0, Solarbio, China) in paraffin or frozen sections. The sections were permeated with 0.2% Triton X-100 and blocked with 10% goat serum for 30 min at 37 °C. Besides, GC-1spg cells were fixed with 4% PFA at room temperature for 30 min, permeated with 0.2% Triton X-100, and blocked with 10% goat serum. The sections or cells were incubated with the primary antibody overnight at 4 °C after blocking with goat serum. The secondary antibodies conjugated to fluorescein isothiocyanate (FITC, 1:200; Invitrogen, USA) or tetramethylrhodamine isothiocyanate Fluor (1:200; Invitrogen, USA) were used to test the primary antibody, and the nuclei were stained with DAPI. The image was captured using an LSM900 confocal laser scanning microscope (Zeiss, Oberkochen, Germany). The antibodies used were as follows: γH2AX (1:200; Abcam, UK), PCNA (1:100; Santa, USA), P53 (1:200; CST, USA), SOX9 (1:200; ABclonal, USA), SCP3 (1:200; Abcam, UK).

### Immunohistochemical staining

For immunohistochemical staining of the testis, the paraffin sections were dewaxed and rehydrated, and 0.01% sodium citrate buffer (pH 6.0, Solarbio, China) was used for antigen repair. The sections were permeated with 0.2% Triton X-100 for 10 min and blocked with 10% goat serum for 30 min at 37 °C. After goat serum removal, the primary antibody was incubated at 4 °C overnight. Then, SP-9000 SPlink Detection Kits (Biotin-Streptavidin HRP Detection Systems, ZSGB-BIO, China) were used to test antibody binding. The antibodies used were as follows: P-cofilin (1:200; CST, USA) and SOX9 (1:200; ABclonal, USA), c-KIT (1:200; Abcam, UK).

### F-actin detection

Phalloidin is a natural toxin purified from the mushroom *Amanita phalloides* to label the F-actin of the cytoskeleton^58^. The mice testes were fixed with 4% paraformaldehyde overnight at 4 °C and suspended in Tissue-Tek OCT Compound (SAKURA, USA). The frozen section experiment was carried out. After washing frozen sections with PBS, phalloidin (1:500, Abcam, USA) was incubated for 40 min at 37 °C to show F-actin, and the nuclei were stained with DAPI. For GC-1spg cells, the cells were fixed with 4% PFA for 20 min and washed with PBS after treatment with Si-RhoGDIα and NSC23766 or Y-27632. Then, phalloidin was also incubated for 40 min at 37 °C. The results were captured using the LSM900 confocal laser scanning microscope (Zeiss, Oberkochen, Germany).

### TUNEL assay

Testis and GC-1spg cell apoptosis was measured using TUNEL assay with an in situ cell death detection kit (Roche, Basel, Switzerland) following the manufacturer’s protocols. Paraffin sections of testicular tissue were dewaxed and rehydrated, then incubated with 3% H_2_O_2_ in methanol solution for 10 min at room temperature, and then subjected to TUNEL staining. After fixing in 4% PFA for 20 min, the GC-1spg cells were incubated with 3% H_2_O_2_ in methanol solution for 10 min, TUNEL incubation was then performed.

### Western blot analysis

Western blot analysis was performed using standard methods. The antibodies used were as follows: RhoGDIα (1:1000; Abcam, USA), P-LIMK (1:1000; CST, USA), LIMK (1:1000; Proteintech, USA), P-cofilin (1:1000; CST, USA), cofilin (1:1000; Proteintech, USA), P53 (1:1000; CST, USA), Rac1 (1:1000; Proteintech, USA), Cdc42 (1:1000; Proteintech, USA), Pak4 (1:1000; Proteintech, USA), Rock1 (1:1000; Proteintech, USA), RhoA (1:1000; Affinity, China) and GAPDH (1:10000; Proteintech, USA). Rac1 and RhoA activity was detected by the Rac1/RhoA Pull-Down Activation Assay Biochem Kit (Cytoskeleton, USA). The Western blot bands were quantified by ImageJ software and normalized to GAPDH.

### GC-1spg cell culture and treatment

GC-1spg cells were cultured in Dulbecco’s modified Eagle’s medium (DMEM, Hyclone Laboratories, USA) supplemented with 10% fetal bovine serum (Gibco, USA), 100 U/mL penicillin and streptomycin. SiRNAs of RhoGDIα (Si-RhoGDIα, 100 nmol/L) were transfected when cells reached 80% confluence. The sequence of siRNA was as follows: Sense of Si‐RhoGDIα-3: 5′‐UCCGGGUGAACAGAGAGAUTT‐3′, antisense 5′‐AUCUCUCUGUUCACCCGGATT‐3′; NC for Si‐RhoGDIα, sense 5′‐UUCUCCGAACGUGUCACGUTT‐3′, antisense 5′‐ACGUGACACGUUCGGAGAATT‐3′. X‐treme GENE siRNA transfection reagent (Roche, Switzerland) was used for the transfection of siRNA into cells following the manufacturer’s protocol. The cells were treated with 10 μM NSC23766 (Aladdin, China) after transfection for 6 h or treated with 140 nM Y-27632 (Aladdin, China) after transfection for 12 h. Immunofluorescence staining, cell viability assay, and TUNEL assay were performed after 24 h treatment with drugs, and total protein was extracted after 48 h treatment of drugs.

### Cell viability assay

The cell viability was tested using MTT assay. The cells were seeded into 96‐well plates with 1 × 10^4^ cells/well. After treatment in different experimental groups, 20 μL of MTT (Sigma‐Aldrich, USA) solution was added to each well and incubated at 37 °C for 4 h. Then, the supernatant was discarded, and 150 μL of dimethyl sulfoxide (DMSO) was added to dissolve the formazan crystals. Finally, the optical density of each well was measured at 490 nm.

### Sperm acrosome detection

The FITC conjugate PNA (sigama, USA) was used to assess acrosomes^59^. In brief, sperms were released from the cauda epididymis and capacitated for 30 min. After washing the sperms with PBS, they were coated on slides and air-dried. The sperms were fixed with 4% PFA. After washing with PBS, the sperm acrosomes were stained with PNA (15 ug/mL in PBS) for 40 min at 37 °C. For acrosomes of the testis, the testicular paraffin sections were dewaxed and rehydrated, and then PNA was stained at 37 °C for 40 min. DAPI was used to stain the nuclei. As for the spontaneous AR frequency test, the sperms from epididymis were incubated in a TYH medium (Nanjing Aibei Biotechnology Co., Nanjing, China) that was balanced at 37 °C under 5% CO_2_ overnight and capacitation for 50 min. The spontaneous AR frequency was shown by the ratio of PNA-negative sperms to DAPI-positive sperms. More than 200 sperms were counted for each mouse.

### Transmission electronic microscopy

Sperm cells were released from the caudal epididymis for 30 min at 37 °C. After washing with PBS, sperm cells were centrifuged for 10 min at 3000 rpm. Then the clump of sperm cells was immobilized with glutaraldehyde and osmium, infused in 10% gelatin, dehydrated with sucrose, and frozen in liquid nitrogen. Frozen slicing (50 nm) was performed using a cryo-ultramicrotome (EM FC7; Leica, Germany) and observed using a JEM-1200EX microscope (JEOL, Japan) following the manufacturer’s protocol.

### Scanning electron microscopy

Sperm samples from the cauda epididymis of *RhoGDIα*^*+/+*^ and *RhoGDIα*^*−/−*^ mice were washed with PBS, spread onto slides and air dried. The samples were fixed with 2.5% glutaraldehyde and post-fixed in 1% osmium tetroxide for 2 h before dehydration in 25%, 50%, 75%, 90%, and 100% gradient ethanol. Then, they were subjected to critical point drying in an Autosamdri-815A (Tousimis, USA). The samples were mounted with gold, and a JEOL 7000 field emission gun SEM (QUANTA 250 FEG, FEI, USA) was used to observe the morphology of sperms.

### Statistics and reproducibility

All the experimental data were presented as mean ± SD, Student’s *t*-test was used for comparing data, and *P* < 0.05 was considered statistically. The graphs were produced by GraphPad Prim software. At least three replicates were performed to ensure reproducibility. Sample sizes and the defined replicates are showed in the figure legend.

### Reporting summary

Further information on research design is available in the Nature Portfolio Reporting Summary linked to this article.

## Supplementary information

Supplementary Information

Description of Additional Supplementary Files

Supplementary Data 1

Reporting Summary

## Data Availability

All data generated or analysed during this study are included in this published article [the source data behind the graphs are available in Supplementary Data 1]. The original blots for the paper are showed in Supplementary Fig. 9. And further inquiries can be directed to the corresponding author.
