# Supplementary material for: RhoGDIα regulates spermatogenesis through Rac1/cofilin/F-actin signaling
Source: Commun Biol. 2023 Feb 23;6:214. doi: 10.1038/s42003-023-04579-7 (PMC9950379; doi:10.1038/s42003-023-04579-7)
Supplement: Supplementary file 1 — Supplementary Information [file 42003_2023_4579_MOESM1_ESM.pdf]

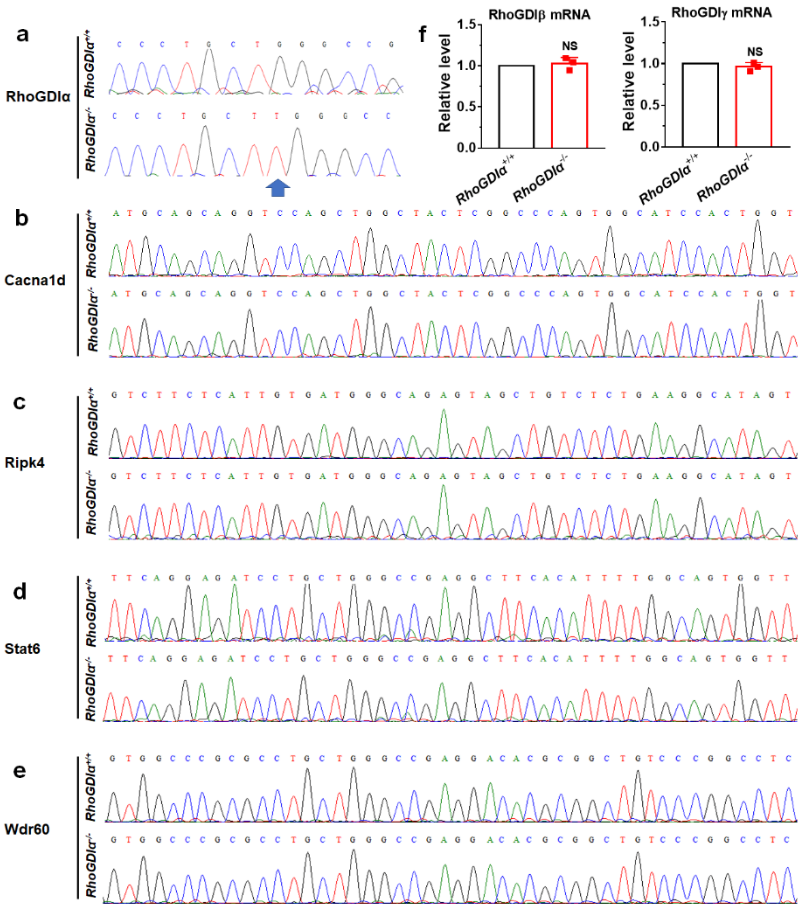

Supplementary Fig. 1 DNA sequencing results of *RhoGDIα*$^{+/+}$ and *RhoGDIα*$^{-/-}$ mice. **a** A base was inserted into the second exon of RhoGDIα to generate frame-shift mutations in *RhoGDIα*$^{-/-}$ mice. **b-e** DNA sequencing results of CACNA1D, RIPK4, STAT6, and WDR60 genes in *RhoGDIα*$^{+/+}$ and *RhoGDIα*$^{-/-}$ mice. **f** The mRNA levels of RhoGDIβ and RhoGDIγ. n = 3 biologically independent animals. NS = non-significant vs *RhoGDIα*$^{+/+}$. Data are presented as the mean ± standard error.

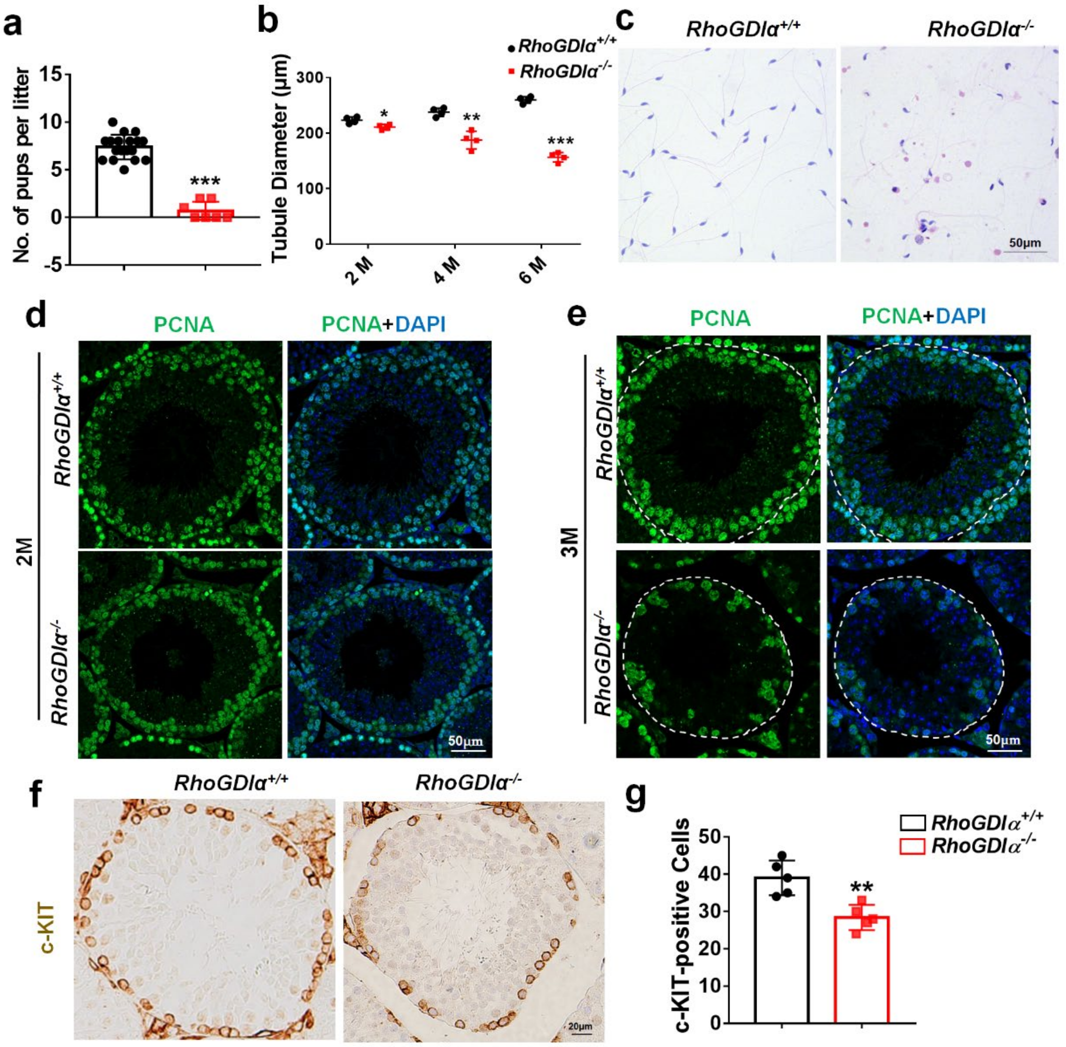

Supplementary Fig. 2 Severe testicular injury occurred in *RhoGDIα*$^{-/-}$ mice. a Litter size of *RhoGDIα*$^{-/-}$ female mice after three months of caging with WT males. n = 6 biologically independent female animals. **b** Diameters of round seminiferous tubules of *RhoGDIα*$^{+/+}$ and *RhoGDIα*$^{-/-}$ mice at 2 months (2M), 4 months (4M), and 6 months (6M). n = 4 biologically independent animals. **c** H&E staining of sperms from 3-month-old *RhoGDIα*$^{+/+}$ and *RhoGDIα*$^{-/-}$ mice. Scar bar =50 μm. **d, e** Immunofluorescence staining for PCNA in testis of 2 months and 3 months of age mouse. Scar bar =50 μm. **f, g** Immunohistochemical results of c-KIT in 3-months mouse testis. Scar bar =20 μm. n = 5 biologically independent animals. *P < 0.05, **P < 0.01, ***P < 0.001 *vs* *RhoGDIα*$^{+/+}$ mice. Data are presented as the mean ± standard error.

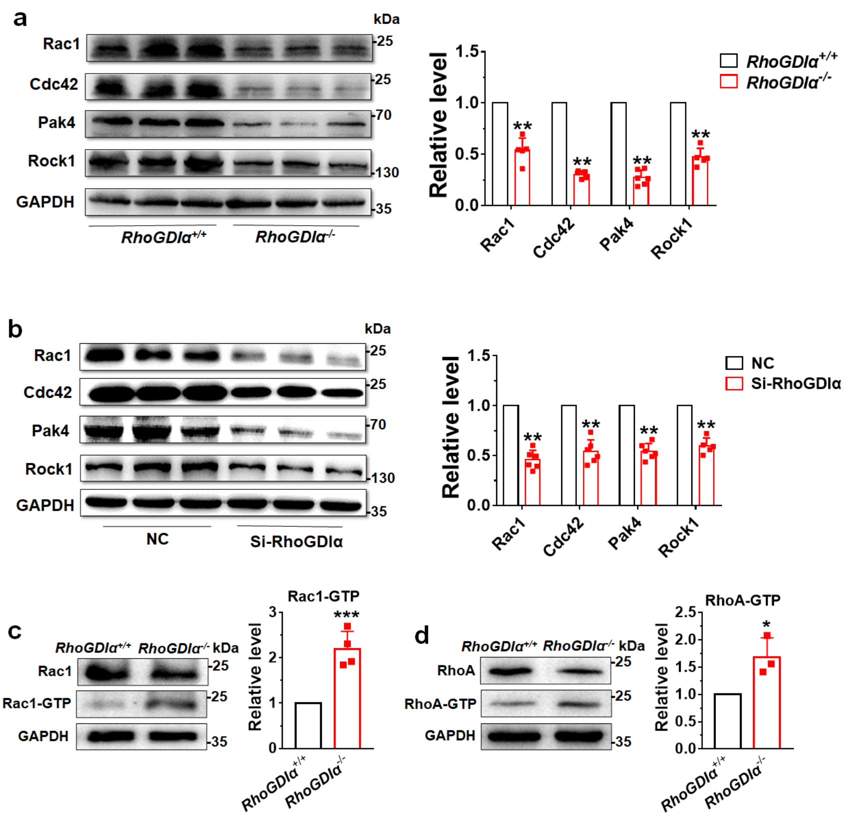

Supplementary Fig. 3 Expression of the Rho GTPase family and its downstream factors decreased after RhoGDIα deletion. a Western blot analysis was used to detect the protein levels of Rac1, Cdc42, Pak4, and Rock1 in *RhoGDIα*$^{+/+}$ and *RhoGDIα*$^{-/-}$ mice testes. **b** Protein expression of Rac1, Cdc42, Pak4, and Rock1 decreased after RhoGDIα knockdown in GC-1spg cells. **a**, **b** n =6 batches of cells. **c, d** The active Rac1 and RhoA were increased in *RhoGDIα*$^{-/-}$ mice testes. n =3 batches of cells. *$P < 0.05$, **$P < 0.01$, **$P < 0.01$ *vs* NC group. Data are presented as the mean ± standard error.

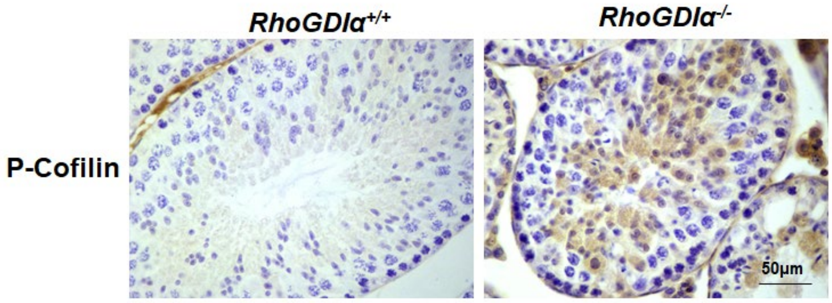

Supplementary Fig. 4 IHC image of P-cofilin expression in *RhoGDIα*$^{+/+}$ and *RhoGDIα*$^{-/-}$ mice testes. Scar bar = 50 μm.

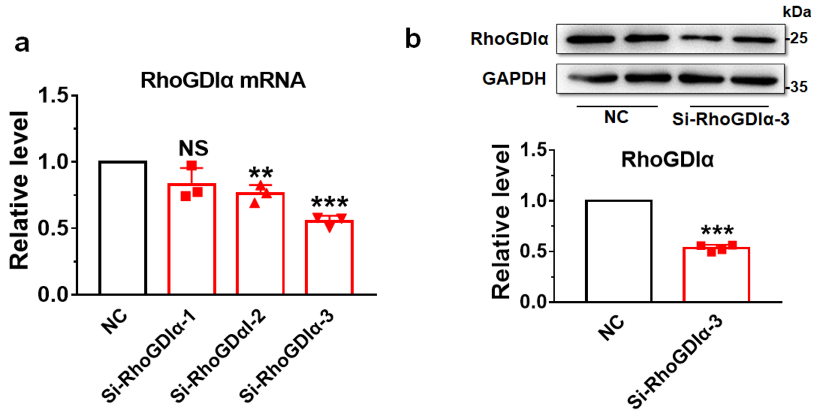

Supplementary Fig. 5 Si-RhoGDIα-3 significantly reduced mRNA and protein levels of RhoGDIα in GC-1spg cells. a Compared with Si-RhoGDIα-1 and Si-RhoGDIα-2 groups, Si-RhoGDIα-3 exerted the best effect to knock down RhoGDIα. n =3 batches of cells. **b** After transfection of Si-RhoGDIα-3, the RhoGDIα protein level decreased to 50% in GC-1spg cells compared with that in the NC group. n =4 batches of cells. $^{**}P < 0.01$, $^{***}P < 0.001$ *vs* NC group. Data are presented as the mean ± standard error.

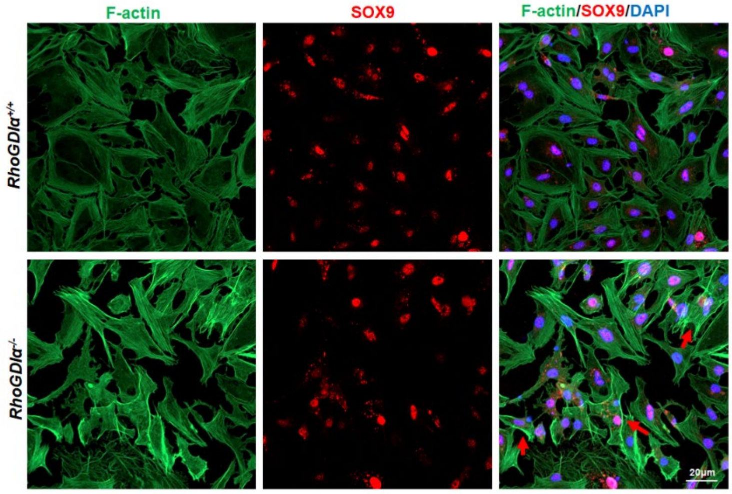

Supplementary Fig. 6 The abnormal F-actin level was detected in SOX9-positive cells in *RhoGDIα*$^{-/-}$ mice (as indicated by red arrows)**.** Scar bar = 20 μm

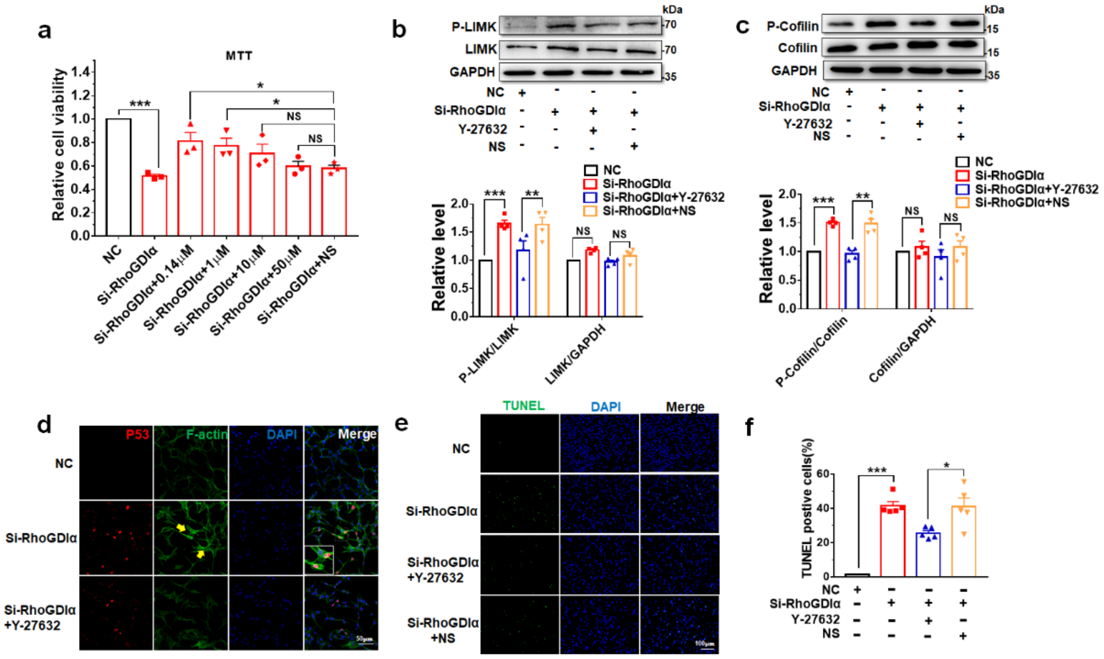

Supplementary Fig. 7 ROCK inhibitor Y-27632 reduced F-actin polymerization and apoptosis in GC-1spg cells under Si-RhoGDIα treatment. a MTT assay was used to test the optimum concentration of the Y-27632 to improve GC-1spg cell viability. n =3 batches of cells. **b** Western blot analysis was used to detect the expression of P-LIMK and LIMK after Y-27632 treatment for 48 h. **c** Y-27632 inhibited the increased phosphorylation of cofilin induced by the knockdown of RhoGDIα. **b**, **c** n = 4 batches of cells. **d** Aggregation of F-actin and the expression of P53 were detected by immunofluorescence. Yellow arrows point to the abnormal aggregation of F-actin. The inside of the solid white box is an enlarged image of the white dashed box. Scar bar = 50 μm. **e, f** TUNEL assay was used to test cell apoptosis after Y-27632 treatment of GC-1spg cells for 24 h. Scar bar = 100 μm. n = 5 batches of cells. ***$P < 0.001$ *vs* NC group, #$P < 0.05$, ##$P < 0.01$ *vs* Si-RhoGDIα group. Data are presented as the mean ± standard error.

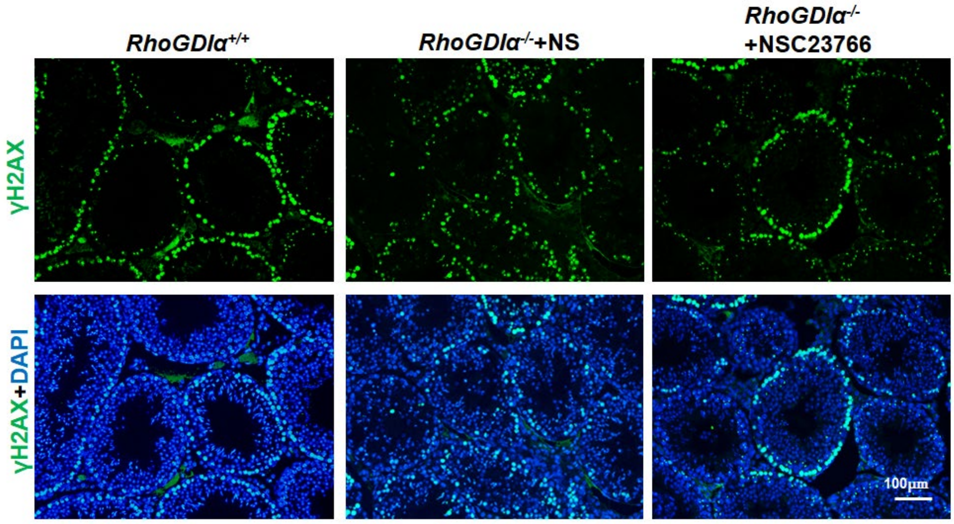

Supplementary Fig. 8 Immunofluorescence results of γH2AX in *RhoGDIα*$^{-/-}$ mouse testes under NSC23766 treatment. Scar bar = 100 μm.

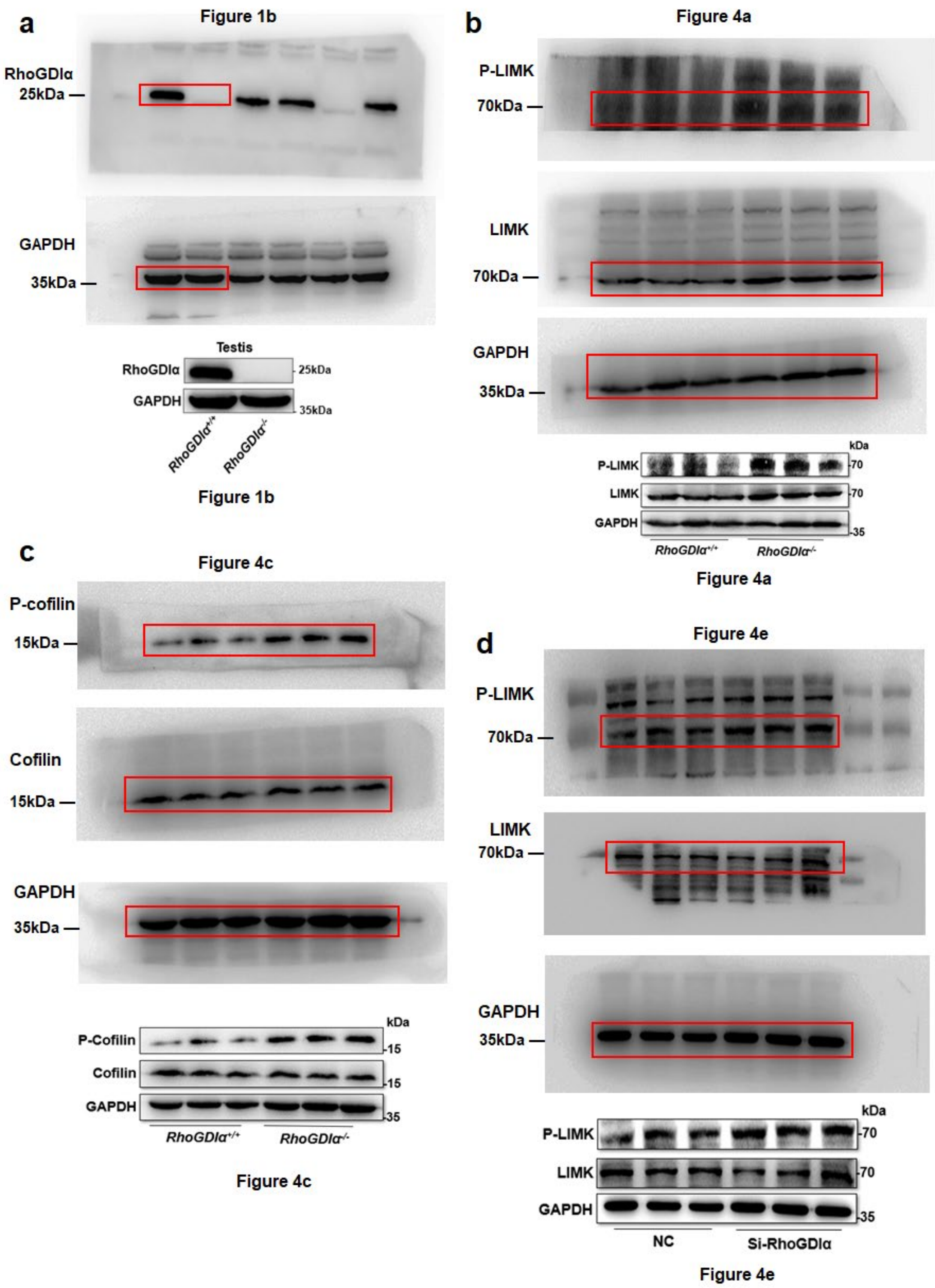

Supplementary Fig. 9 Uncropped scans of Western blot results.

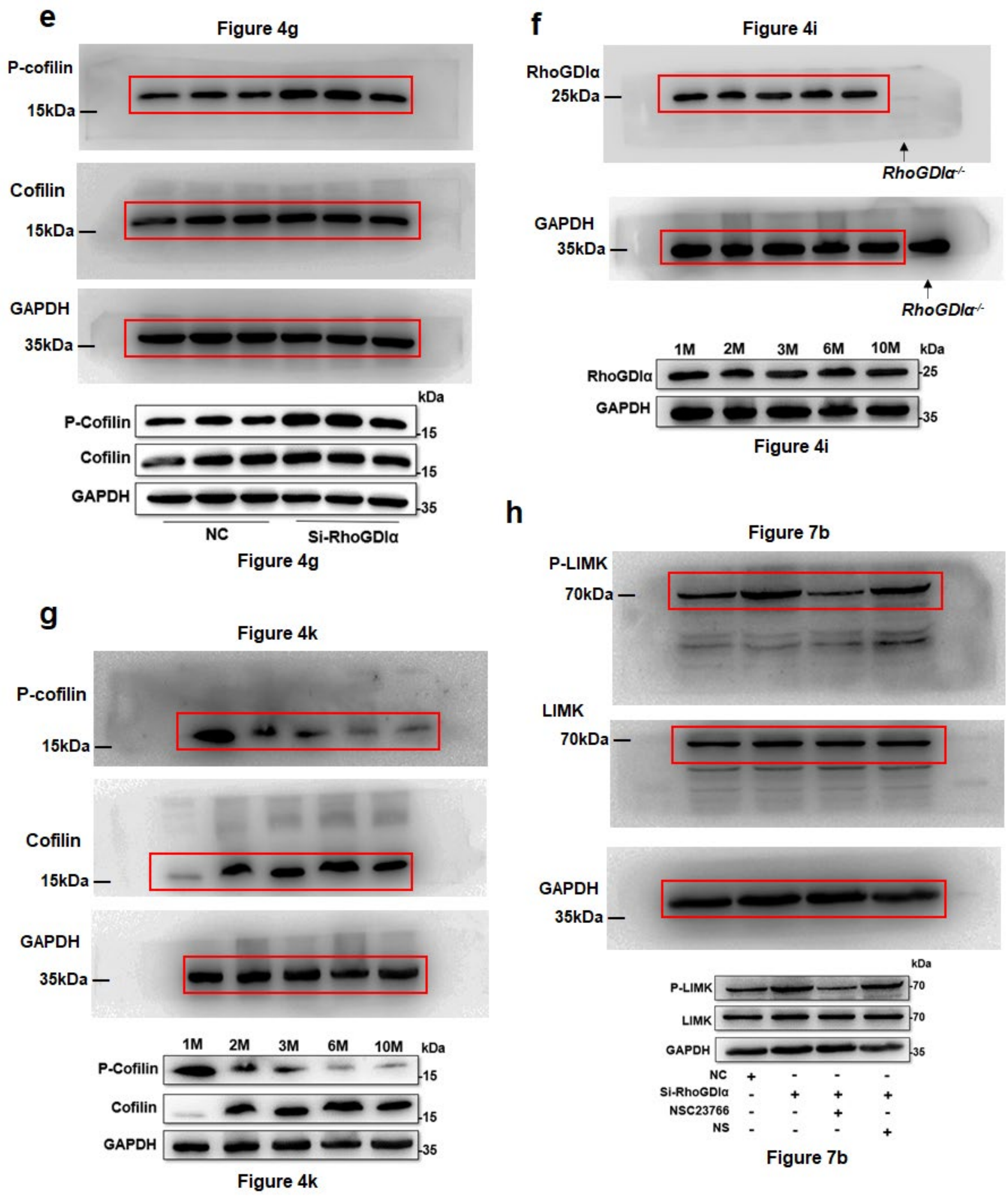

Supplementary Fig. 9 Uncropped scans of Western blot results.

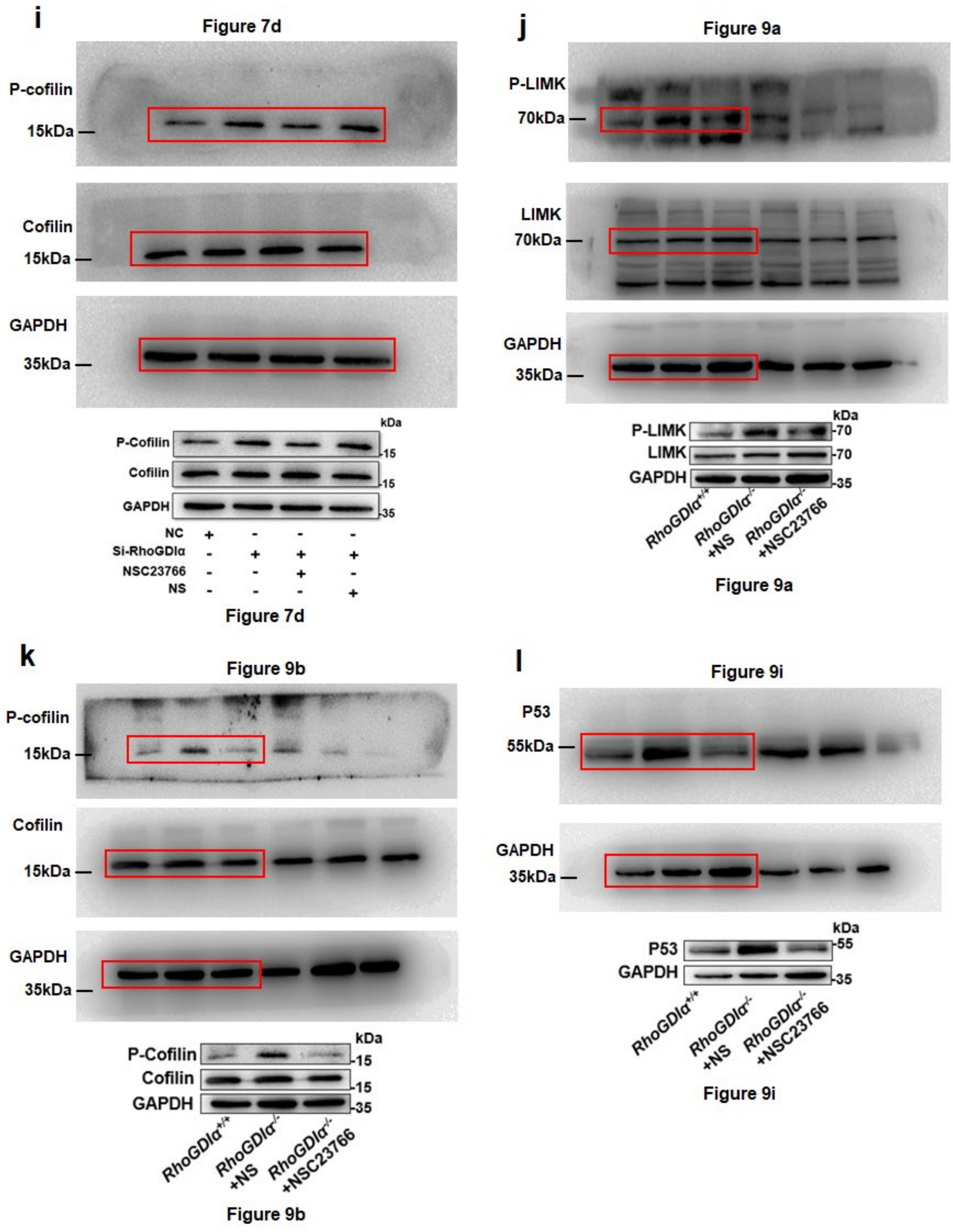

Supplementary Fig. 9 Uncropped scans of Western blot results.
