# Supplementary material for: RhoGDIα regulates spermatogenesis through Rac1/cofilin/F-actin signaling
Source: Commun Biol. 2023 Feb 23;6:214. doi: 10.1038/s42003-023-04579-7 (PMC9950379; doi:10.1038/s42003-023-04579-7)
Supplement: Supplementary file 4 — Reporting Summary [file 42003_2023_4579_MOESM4_ESM.pdf]

nature portfolio

Corresponding author(s): Jiangang Gao

Last updated by author(s): Jan 31, 2023

Reporting Summary

Nature Portfolio wishes to improve the reproducibility of the work that we publish. This form provides structure for consistency and transparency in reporting. For further information on Nature Portfolio policies, see our Editorial Policies and the Editorial Policy Checklist.

Statistics

For all statistical analyses, confirm that the following items are present in the figure legend, table legend, main text, or Methods section.

n/a	Confirmed	
☐	☒	The exact sample size (*n*) for each experimental group/condition, given as a discrete number and unit of measurement
☐	☒	A statement on whether measurements were taken from distinct samples or whether the same sample was measured repeatedly
☐	☒	The statistical test(s) used AND whether they are one- or two-sided<br>*Only common tests should be described solely by name; describe more complex techniques in the Methods section.*
☒	☐	A description of all covariates tested
☐	☒	A description of any assumptions or corrections, such as tests of normality and adjustment for multiple comparisons
☐	☒	A full description of the statistical parameters including central tendency (e.g. means) or other basic estimates (e.g. regression coefficient) AND variation (e.g. standard deviation) or associated estimates of uncertainty (e.g. confidence intervals)
☐	☒	For null hypothesis testing, the test statistic (e.g. *F*, *t*, *r*) with confidence intervals, effect sizes, degrees of freedom and *P* value noted<br>*Give P values as exact values whenever suitable.*
☒	☐	For Bayesian analysis, information on the choice of priors and Markov chain Monte Carlo settings
☒	☐	For hierarchical and complex designs, identification of the appropriate level for tests and full reporting of outcomes
☒	☐	Estimates of effect sizes (e.g. Cohen's *d*, Pearson's *r*), indicating how they were calculated

Our web collection on statistics for biologists contains articles on many of the points above.

Software and code

Policy information about availability of computer code

Data collection	Graphpad Prism version 7.0
Data analysis	Graphpad Prism version 7.0

For manuscripts utilizing custom algorithms or software that are central to the research but not yet described in published literature, software must be made available to editors and reviewers. We strongly encourage code deposition in a community repository (e.g. GitHub). See the Nature Portfolio guidelines for submitting code & software for further information.

Data

Policy information about availability of data

All manuscripts must include a data availability statement. This statement should provide the following information, where applicable:

- Accession codes, unique identifiers, or web links for publicly available datasets
- A description of any restrictions on data availability
- For clinical datasets or third party data, please ensure that the statement adheres to our policy

The data that support the findings of this study are available from the corresponding author upon reasonable request.

Human research participants

Policy information about studies involving human research participants and Sex and Gender in Research.

Reporting on sex and gender	No human research were involved in this study.
Population characteristics	See above.
Recruitment	See above.
Ethics oversight	See above.

Note that full information on the approval of the study protocol must also be provided in the manuscript.

Field-specific reporting

Please select the one below that is the best fit for your research. If you are not sure, read the appropriate sections before making your selection.

☒ Life sciences ☐ Behavioural & social sciences ☐ Ecological, evolutionary & environmental sciences

For a reference copy of the document with all sections, see nature.com/documents/nr-reporting-summary-flat.pdf

Life sciences study design

All studies must disclose on these points even when the disclosure is negative.

Sample size	Experimental sample size were estimated based on our past experience performing similar studies evaluating rescue efficiency in mice and cells.
Data exclusions	There was no data exclusion.
Replication	All studies were conducted in accordance with recently issued NIH guidelines on reproducibility and rigor in scientific research.
Randomization	All data analysis were performed under randomization.
Blinding	Key of sample groups were blinded for analysis.

Reporting for specific materials, systems and methods

We require information from authors about some types of materials, experimental systems and methods used in many studies. Here, indicate whether each material, system or method listed is relevant to your study. If you are not sure if a list item applies to your research, read the appropriate section before selecting a response.

Materials & experimental systems

n/a	Involved in the study	
☐	☒	Antibodies
☐	☒	Eukaryotic cell lines
☒	☐	Palaeontology and archaeology
☐	☒	Animals and other organisms
☒	☐	Clinical data
☒	☐	Dual use research of concern

Methods

n/a	Involved in the study	
☒	☐	ChIP-seq
☒	☐	Flow cytometry
☒	☐	MRI-based neuroimaging

Antibodies

Antibodies used

Anti-RhoGDIα antibody（abcam, ab135252, 2G3, GR313436-10）
Anti-γH2ax antibody（abcam, ab26350, 9F3, GR3427575-1）
Anti-SCP3 antibody（abcam, ab15093, GR3247082-7）
Anti-PCNA antibody（SANTA, sc-56, PC10, B0321）
Anti-SOX9 antibody（ABclonal, A19710, 4000000190）
Anti-C-kit antibody（R&D systems, AF1356, IEO 0421061）
Anti-P-Limk antibody（Cell Signaling Technology, 38415, 7）

Anti-Limk antibody（Proteintech, 9699-1-1AP, 00025781）
Anti-P-cofilin antibody（Cell Signaling Technology, #3313, 77G2, 7）
Anti-Cofilin antibody（Proteintech, 66057-1-Ig, 10003113 ）
Anti-GAPDH antibody（abcam, ab135252, 00069969）
Anti-P53 antibody（Cell Signaling Technology, #2524, 1C12, 15）
Anti-Rac1 antibody（Proteintech, 24072-1-AP, 00042560）
Anti-RhoA antibody（Affinity, #AF6352, #63k3835）
Anti-Cdc42 antibody（Proteintech, 10155-1-AP, 00041192）
Anti-Pak4 antibody（Proteintech, 14685-1-AP, 00006742）
Anti-Rock1 antibody（Proteintech, 21850-1-AP, 00052277）

Validation: We have specifically validated antibodies.

Eukaryotic cell lines

Policy information about cell lines and Sex and Gender in Research

Cell line source(s)	GC-1spg cells were obteined from Procell Life Science&Technology Co,.Ltd.
Authentication	We declare that we have not authenticated the cells purchased from PLST.
Mycoplasma contamination	We confirm that GC-1spg cells tested negative for mycoplasma contamination.
Commonly misidentified lines (See ICLAC register)	None

Animals and other research organisms

Policy information about studies involving animals; ARRIVE guidelines recommended for reporting animal research, and Sex and Gender in Research

Laboratory animals	The experimental animals of this study were C57BL/6 mice, aged from 14 days to 6 months.
Wild animals	The study did not involve wild animals.
Reporting on sex	This study is about male sterility of mice, and the reproductive ability of female mice has also been tested, which has been mentioned in the manuscript. Sex is determined primarily by the mouse's reproductive system.
Field-collected samples	The study did not involve samples collected from the field.
Ethics oversight	All animal experiments are conducted in accordance with the approval of the Ethics Committee of Shandong University (Jinan, China), and animal management is strictly in accordance with the Animal Ethics Standards of Shandong University.

Note that full information on the approval of the study protocol must also be provided in the manuscript.
